# Initiation of anti-osteoporosis medication following hip fracture in older adults: a systematic review and thematic synthesis of qualitative studies from patient and healthcare professional perspectives

**DOI:** 10.1093/ageing/afaf237

**Published:** 2025-08-21

**Authors:** Sabine de Jong-Holthuijsen, Willeke M Ravensbergen, Wilco P Achterberg, Natasha M Appelman-Dijkstra, Jacobijn Gussekloo, Rosalinde K E Poortvliet

**Affiliations:** Dept of Public Health and Primary Care, Leiden University Medical Center, Leiden, the Netherlands; LUMC Center for Medicine for Older People (LCO), Leiden University Medical Center, Leiden, the Netherlands; Dept of Public Health and Primary Care, Leiden University Medical Center, Leiden, the Netherlands; LUMC Center for Medicine for Older People (LCO), Leiden University Medical Center, Leiden, the Netherlands; Dept of Public Health and Primary Care, Leiden University Medical Center, Leiden, the Netherlands; LUMC Center for Medicine for Older People (LCO), Leiden University Medical Center, Leiden, the Netherlands; Dept of Internal Medicine, Division of Endocrinology and Leiden Center for Bone Quality, Leiden University Medical Center, Leiden, the Netherlands; Dept of Public Health and Primary Care, Leiden University Medical Center, Leiden, the Netherlands; LUMC Center for Medicine for Older People (LCO), Leiden University Medical Center, Leiden, the Netherlands; Dept of Internal Medicine, Section of Gerontology and Geriatrics, Leiden University Medical Center, Leiden, the Netherlands; Dept of Public Health and Primary Care, Leiden University Medical Center, Leiden, the Netherlands; LUMC Center for Medicine for Older People (LCO), Leiden University Medical Center, Leiden, the Netherlands

**Keywords:** hip fracture, osteoporosis, anti-osteoporosis medication, decision-making, qualitative, research, older people

## Abstract

**Background:**

Pharmacological osteoporosis treatment is recommended for secondary fracture prevention in older patients following a hip fracture. However, not all eligible patients receive anti-osteoporosis medication (AOMs). Understanding the decision-making process regarding treatment initiation at patient level may help explain low prescription rates and offer novel solutions beyond organisational initiatives.

**Objective:**

This systematic review aimed to synthesise data from qualitative studies on the initiation of AOMs after a hip fracture in old age, to (i) explore the experiences and preferences of patients and healthcare professionals in the decision-making process, and (ii) clarify if this explains low prescription rates.

**Methods:**

A systematic search in seven medical databases identified qualitative publications on the initiation of AOMs in older hip fracture patients. Thematic synthesis was applied, with the CASP checklist and GRADE-CERQual approach enhancing review’s rigour.

**Results:**

Twenty studies were included, revealing two main themes with eight subthemes. The ‘addressing’ step illustrates that addressing osteoporosis treatment is not self-evident and depends on specialty-specific responsibilities, knowledge and capability, commitment to identifying eligible patients, and the perceived importance and feasibility of treatment. The ‘discussing’ step highlights the need for patient education, patient perceptiveness, making sense, a patient-centred approach, and patient choice, indicating that discussing treatment is not a clear-cut path.

**Conclusions:**

AOM initiation after hip fracture in old age is shaped by a two-step decision-making process of (i) addressing treatment and (ii) discussing treatment—which may partly explain low prescription rates. Beyond organisational strategies, promoting education and awareness may strengthen professional initiative and patient engagement.

## Key Points

- Pharmacological treatment of osteoporosis is recommended following a hip fracture in older patients; however, only a minority of patients receive anti-osteoporosis medication.
- Initiating anti-osteoporosis medication after a hip fracture in old age depends on the attitudes and decisions of patients and healthcare professionals, as reflected in the two-step decision-making process: (i) addressing treatment and (ii) discussing treatment.
- Addressing osteoporosis treatment is not self-evident for healthcare professionals and depends on specialty-specific responsibilities, knowledge and capability, commitment to identifying eligible patients, and the perceived importance and feasibility of treatment from both a clinical and patient perspective.
- Discussing treatment is not a clear-cut path for both healthcare professionals and patients, and requires raising awareness through patient education, patient perceptiveness, the feeling that it makes sense, a patient-centred approach, and patient choice in osteoporosis treatment.
- Beyond organisational strategies, promoting education and awareness concerning post-hip fracture osteoporosis care may strengthen professional initiative and patient engagement.

## Introduction

Worldwide, over 10 million hip fractures occur annually in people aged 55 and over, and this number is expected to double by 2050 due to an ageing society and increased life expectancy [1, 2]. As stated in the SCOPE 2021 report, the lifetime probability of hip fracture at the age of 50 years is 4.8% for men and 13.8% for women in the United Kingdom, and 5.4% for men and 12.5% for women in the Netherlands [3]. Hip fractures have a major impact on individuals and society in terms of fracture-associated mortality, morbidity, functional outcomes and related healthcare costs.

Subsequent fractures significantly contribute to these detrimental outcomes, making their prevention a critical aspect of post-hip fracture care. A long-term follow-up study indicated that 35% of the hip-fracture patients experienced a subsequent fracture, with 45% of these occurring within the first year following the initial hip fracture [4]. In 2007, Lyles *et al*. demonstrated that treatment with anti-osteoporosis medication (AOMs) reduced mortality after a hip fracture [5]. More recently, this finding has been supported by real-life data, indicating that initiating bisphosphonates during the initial fracture hospitalisation reduces both mortality rates and the risk of subsequent fractures [6]. The initiation of AOMs, therefore, is strongly recommended for all older patients with a life expectancy of at least one year after a hip fracture [7–9]. Early initiation of AOMs is particularly effective in reducing subsequent fractures in (frail) older patients, highlighting the importance of timely intervention [10].

However, hip fracture patients are often not prescribed AOMs [11]. In Europe, estimates indicate that 49% (UK) to 84% (Sweden) of hip fracture patients do not receive AOMs in the first year after a hip fracture, and these numbers have remained within this range for years [12].

Several causes might contribute to the low prescribing rates of AOMs after a hip fracture in old age, such as organisational barriers [13]. Organisational aspects play a significant role in post-fracture management of osteoporosis. A key example is the widespread success of implementing structured care models, such as the integration of Fracture Liaison Services (FLSs), in increasing prescription rates of AOMs following a fracture [14, 15]. However, improving organisational aspects alone may not be sufficient to enhance prescription rates, as patient-related factors—including sex, age and comorbidities—appear to influence the initiation of AOMs, particularly in older patients with fragility fractures [16]. Fu *et al*. have demonstrated that patient-factors play an important role in the process of decision-making regarding initiating AOMs in older individuals [17]. Moreover, at the level of the individual patient and healthcare professional, information is lacking on the process of decision-making regarding the initiation of AOMs after a hip fracture. Novel insights into the decision-making process, gained through qualitative research, may help explain the association between patient-related factors and undertreatment. These insights could offer potential solutions to increase prescription rates, complementing existing organisational initiatives. Qualitative research can provide insight into people’s thoughts, motivations and values, allowing an examination of a phenomenon and its context. Combining the insights of multiple qualitative studies through a qualitative systematic review may offer more insight than the sum of the studies individually [18]. Our aim is to perform a systematic review of qualitative studies by accumulating and analysing all the available qualitative evidence at the patient level on the initiation of AOMs after a hip fracture in old age, in order to: (i) explore the experiences and preferences of both patients and healthcare professionals with regard to the process of decision-making, and (ii) clarify whether, and if applicable, how the process of decision-making provides potential explanations for the low prescribing rates of AOMs.

## Methods

We used the ENTREQ (Enhancing transparency in reporting the synthesis of qualitative research) checklist [19] (Table S1) to enhance transparency in reporting the qualitative thematic synthesis of this review. The protocol of this review was registered in the PROSPERO database: CRD42024550702.

### Inclusion criteria

The inclusion criteria encompassed all types of written, published qualitative studies that contained data on post-fracture pharmacological treatment of osteoporosis for older patients (i.e. patients aged 60 year and above) with a low-energy hip fracture. Data had to be collected via interviews, focus groups or field or participant observations. To prevent cultural and linguistic bias, non-English articles were excluded to avoid the potential loss of the original interpretation during translation. For healthcare professionals, we focused on qualitative data regarding osteoporosis care for older patients with a low-energy hip or fragility fracture. The broader fragility fracture population was included at a later stage in the process of thematic analyses. Since the themes initially identified in the hip fracture population also emerged within the broader fragility fracture population, we assumed that underlying statements were particularly relevant to hip fracture patients, as they constitute a substantial subgroup within the fragility fracture population. For patients, we exclusively focused on qualitative data concerning older patients with a hip fracture. Consequently, studies covering a mixed population were excluded if relevant qualitative data could not be linked to a hip fracture patient. Since we focused on the clinical process of decision-making regarding the initiation of AOMs at the patient level, studies that solely focused on contextual factors, e.g. organisational care aspects, were excluded as these contextual factors could not be linked to the (results of the) decision-making process.

### Search strategy

We conducted a comprehensive systematic search on 31 July 2023 in seven electronic databases: PubMed, Embase, Web of Science, Cochrane Library, Emcare, PsycINFO, and Academic Search Premier, and updated this search on 7 February 2025. The search strategy included synonyms for ‘(osteoporotic) hip fracture’ and ‘qualitative methods’ and was developed together with a specialised medical librarian. Grey literature, such as meeting abstracts, was not included (Appendix 2).

### Data screening and study selection

After duplicates were removed, both S.d.J-H and W.M.R. independently screened and selected all articles sequentially based on titles and abstracts to assess whether these articles potentially met the criteria for inclusion. Articles that potentially met the inclusion criteria underwent full-text review by S.d.J-H and W.M.R. to assess whether they met eligibility criteria (Appendix 2). References from included articles and relevant review articles were cross-checked for additional eligible articles. Conflicts in assessment were discussed regularly to reach consensus, and if necessary, a third researcher (R.K.E.P.) was consulted to make the final decision about inclusion or exclusion. Detailed information on the study selection is provided in Appendix 2.

### Data extraction, quality assessment

Data on study characteristics, such as demographics of the study population and the methods of data collection used, were extracted by S.d.J-H, and checked for accuracy by W.M.R. The Critical Appraisal Skills Programme (CASP) checklist [20] was used by both S.d.J-H and W.M.R. independently to assess the methodological quality of the included studies to improve the rigour of the review, as endorsed by the Cochrane Institute and the World Health Organisation [21]. Conflicts in assessment were discussed to reach consensus (Table S2).

### Data synthesis

A systematic approach was used for the thematic synthesis by employing qualitative data analysis software Atlas.ti (version 24). Thematic analysis was used to analyse and compare the data of individual healthcare professionals and patients across different studies to identify overarching themes and concepts, as described by Braun and Clarke [22, 23]. First, for data extraction, the included articles were grouped based on statements of healthcare professionals regarding hip fracture patients or fragility fracture patients (group 1) or statements of hip fracture patients (group 2). Second, the articles were read multiple times by S.d.J-H and W.M.R. to become familiar with the data and identify extractable data. Third, all relevant data in the results and discussion sections were highlighted and coded line by line by S.d.J-H, using inductive coding. Five articles were independently coded by W.M.R. Fourth, the codes were discussed and clustered by S.d.J-H, W.M.R. and R.K.E.P. to develop a code tree and identify initial main themes and subthemes. Fifth, one author (S.d.J-H) completed the coding using the code tree, discussing the identified main themes and subthemes regularly with W.M.R., R.K.E.P. and J.G. until no new themes arose, following the iterative process of inductive thematic analysis. Sixth, the main themes, subthemes and concepts were discussed for refinement within the multidisciplinary research team.

### Confidence in findings

The Confidence in the Evidence from Reviews of Qualitative Research (GRADE-CERQual) approach was used to assess the level of confidence in the synthesised findings from this review (Table S3) [24].

### Reflexivity

We acknowledge that the personal and professional background of individual researchers can influence the interpretation of data in a qualitative systematic review [25]. To ensure a broad perspective on the data, a multidisciplinary group of authors was assembled for this review. Throughout the qualitative systematic review, we engaged in reflexivity to examine our own biases and potential influence on the study. Recognising our professional backgrounds in primary care, internal medicine, and geriatrics, we held regular team discussions to incorporate multiple viewpoints, thereby enhancing the credibility of our findings. Our medical backgrounds may have shaped our interpretation of the data, as our experiences and training influence how we perceived and analysed the participants’ responses. By acknowledging this, we aimed to remain transparent and critical in our approach, providing a nuanced understanding of the decision-making process regarding the initiation of AOMs after a hip fracture.

## Results

### Study selection and characteristics

**Figure 1:**
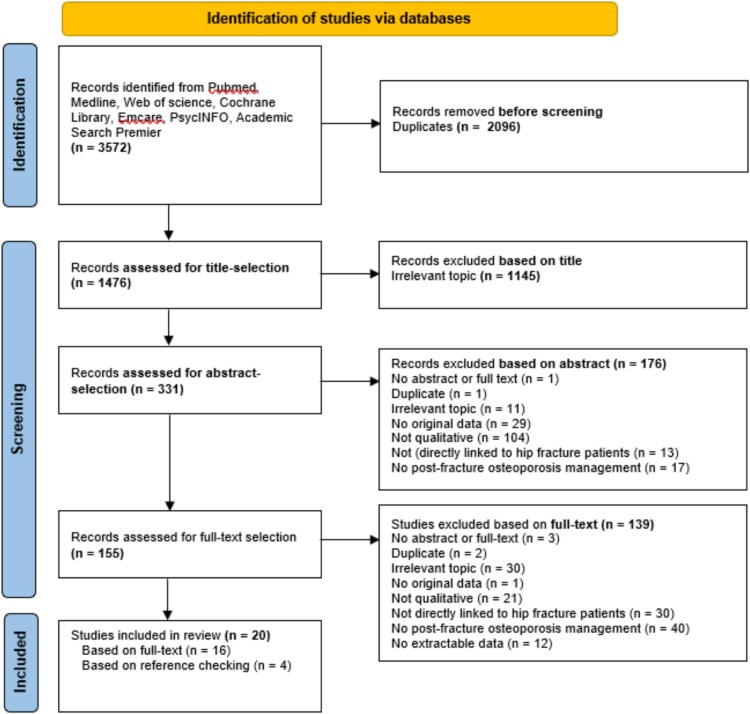
PRISMA flowchart.

We identified 1476 studies, of which 20 studies were included in the analyses for this review (Table 1). The most common reason for exclusion was ‘irrelevant topic’ (Figure 1). In the 20 included studies, qualitative data were collected via semi-structured interviews (*n* = 12), focus groups (*n* = 4) or a combination of interviews and field or participant observations (*n* = 2), or interviews and focus groups (*n* = 2). The perspective of healthcare professionals on osteoporosis care after a hip fracture were extracted from 17 studies, with three studies focusing on the hip fracture population and 14 studies on a mixed population. All extracted data were directly linked to either patients with a hip or with a fragility fracture. The patient perspective on osteoporosis care after a hip fracture was drawn from five studies, with all data explicitly linked to a hip fracture patient.

**Table 1:** Summary of characteristics of the included studies.

First author	Country	Population (number of participants)	Care setting	Focus	Qualitative data collection	Primary aim
Armstrong E 2022 [38]	China, India, The Philippines, Thailand, Vietnam	Healthcare professionals (*n* = 35)	Hospital	Hip fracture patients	Semi-structured interviews	To identify barriers to, and enablers of, evidence-informed hip fracture care in LMICs, and to determine if the Blue Book strategies and standards are applicable to these settings.
Bennett MJ 2023 [34]	Australia	Healthcare professionals (*n* = 18) Patients (*n* = 7); ‘not included in review’	Hospital, primary care	Post-fracture patients	Semi-structured interviews	To map current service processes and integration factors influencing long-term postclinic care, identifying barriers, supports, and opportunities for seamless healthcare following fragility fracture.
Bishop S 2023 [35]	United Kingdom	Healthcare professionals (*n* = 23)^a^	Hospital, primary care	Patients with osteoporosis	Semi-structured interviews	To investigates clinicians’ experiences of prescribing different forms of bisphosphonate medication, focusing on how treatment decisions are made in practice and how this relates to ongoing processes of treatment and care.
Bullock L 2024 [33]	United Kingdom	Healthcare professionals (*n* = 16) Patients (*n* = 4); ‘not included in review’	Hospital, primary care	Post-fracture patients	Focus groups	To explore current practice in relation to communication about osteoporosis medicine, anticipated barriers to, and facilitators of, using a computerised osteoporosis DST, and perceived training needs.
Cheah MH 2024 [30]	Malaysia	Healthcare professionals (*n* = 30)	Hospital	Post-fracture patients	Semi-structured interviews	To explore the views of healthcare professionals regarding the barriers and facilitators for a FLS in Malaysia, especially to facilitate its implementation in this tertiary hospital.
Drew S 2016 [36]	United Kingdom	Healthcare professionals (*n* = 43)	Hospital	Hip fracture patients	Semi-structured interviews	To identify healthcare professionals’ views on effective care for prevention of secondary fracture after hip fracture.
Edwards BJ 2012 [49]	United States of America	Healthcare professionals (*n* = 24^)a^ Patients (*n* = 36); ‘not included in review’	Hospital	Patients with osteoporosis	Focus groups	To utilise all aspects of the chronic care model in order to develop patient-centred, evidence-based recommendations to improve osteoporosis care.
Feldstein AC 2008 [26]	United States of America	Healthcare professionals (*n* = 66^)a^ Patients (*n* = 10)^b^	Hospital, primary care	Patients with fracture	In-depth interviews and focus groups	To use the findings reported here to improve the outreach program in the future, with the long-term goal of improving osteoporosis care and fracture prevention.
Guillemot JR 2024 [37]	France, United Kingdom	Healthcare professionals (*n* = 35)^a^	Hospital, primary care	Patients with osteoporosis	Semi-structured interviews	To unravel the intricacies of medical decision making in geriatric care, offering insights into the evolving landscape of healthcare policy and practice, which in turn can help reduce futile biomedical research.
Jensen CM 2017 [39]	Denmark	Patients (*n* = 10) Healthcare professionals (*n* = 15) Relatives (*n* = 4); ‘not included in review’	Hospital	Hip fracture patients	Field observations and semi-structured interviews	To describe experiences of patients with a hip fracture and explore if the patients felt empowered and able to perform self-care in pathways with short time stay in hospital (STSH).
Jensen CM 2019 [40]	Denmark	Patients (*n* = 25)	Hospital	Hip fracture patients	Narrative interviews and participant observation	To examine whether a tele-health solution in the form of an app can support self-care and empowerment of patients following a fragility fracture of the hip and a short length of hospital stay.
Lerner ET 2025 [31]	United States of America	Healthcare professionals (*n* = 17)	Skilled nursing facility	Patients with fracture	Semi-structured interviews	To understand SNF providers’ perspectives on osteoporosis treatment and to receive feedback on a pocket card to guide osteoporosis treatment in the post-acute care setting.
Merle B 2019 [48]	France	Healthcare professionals (*n* = 16)^a^	Primary care	Patients with osteoporosis	Semi-structured interviews	To explore the knowledge and representations of French GPs regarding OP and its prevention, attitudes related to the use of guidelines, perceived barriers to OP care, and suggestions for improvement.
Narayanasamy M 2022 [51]	United Kingdom	Patients (*n* = 78)^b^	Hospital	Patients with osteoporosis	Semi-structured interviews	To provide insight into the acceptability and engagement of both oral and intravenous bisphosphonate treatments for patients with osteoporosis who are at risk of fragility fractures.
Otmar R 2012 [32]	Canada	Healthcare professionals (*n* = 16)	Primary care	Patients with osteoporosis	Semi-structured interviews and focus groups	To investigate barriers, enablers, and other factors influencing the investigation and management of osteoporosis using a qualitative approach.
Salminen H 2019 [29]	Sweden	Healthcare professionals (*n* = 17)^a^	Primary care	Patients with osteoporosis	Focus groups	To explore primary care physicians’ views on osteoporosis management in primary care.
Tahmasbi F 2024 [50]	Iran	Healthcare professionals (*n* = 13)	Hospital	Post-fracture population	Semi-structured interviews	To provide a comprehensive overview of the current status of FLS implementation in Iran, applying the essential elements outlined in the FLS framework.
Unson CG 2003 [52]	United States of America	Patients (*n* = 45)^b^	Primary care	Patients with osteoporosis	Focus groups	To examine how beliefs about medication and four osteoporosis treatments influenced treatment selection and adherence.
Verdonck C 2023 [28]	Belgium	Healthcare professionals (*n* = 13)^a^	Primary care	Patients with osteoporosis	Semi-structured interviews	To analyse underlying mechanisms hampering Flemish PCPs to fully commit to osteoporosis care.
Wozniak LA 2020 [27]	Canada	Healthcare professionals (*n* = 33)	Hospital	Fragility fracture patients	Semi-structured interviews	To understand the context in which a FLS is implemented, including readiness to implement change as well as facilitators and barriers to implementation.

^a^only data regarding fragility fracture patients included in review,

^b^only data of identifiable hip fracture patients included in review

### Participant characteristics

The included studies were published between 2003 and 2025 and were conducted in the United Kingdom (*n* = 5), United States of America (*n* = 4), Canada (*n* = 2), Denmark (*n* = 2), France (*n* = 2), Australia (*n* = 1), Belgium (*n* = 1), China, India, Thailand, the Philippines and Vietnam (*n* = 1), Iran (*n* = 1), Malaysia (*n* = 1) and Sweden (*n* = 1). Some studies were conducted in multiple countries.

### Methodological quality of included studies

The methodological quality of most included studies was rigorous, as indicated by the CASP assessment (Table S2). The most common methodological limitations were a lack of information on authors’ reflexivity (all 20 studies) and the chosen research design (*n* = 10) and recruitment strategy (*n* = 6), respectively. The most common methodological strengths were an adequately described rationale for the chosen qualitative methodology and added value of the study (all 20 studies), a clear statement of the research aims (*n* = 19), a description of consideration of ethical issues (*n* = 19), and a rationale for data collection (*n* = 18). No articles were excluded based on the CASP assessment.

### Confidence in findings

During the analysis and interpretation of the data, we undertook a systematic process of reflection and evaluation using the GRADE-Cerqual approach. This enabled us to assess the level of confidence in the synthesised findings in relation to the underlying qualitative data and the context in which it was generated. In doing so, we carefully considered the contribution of both patient and healthcare professional data, with particular attention to data derived from mixed populations in the case of healthcare professionals. Less significance was attributed to such data compared to that derived exclusively from the hip fracture population. A detailed overview of this approach is provided in Table S3.

### Thematic synthesis

During the process of thematic analysis, we identified two main themes encompassing eight subthemes (Table 2, Table 3). The main themes are two distinct decision-making steps that emerged from the data, in chronological order: (i) The ‘addressing’ step, (ii) The ‘discussing’ step. The main findings of this review illustrate that addressing pharmacological treatment of osteoporosis after a hip fracture is not self-evident (Main theme I, The ‘addressing’ step) and discussing the initiation of AOMs is not a clear-cut path (Main theme II, The ‘discussing’ step).

### Main theme I: the addressing step—addressing pharmacological treatment of osteoporosis is not self-evident

#### Subtheme I.1 responsibility, knowledge and capability

When characterising osteoporosis care after a hip fracture, healthcare professionals emphasised the multidisciplinary and transitional, acute-to-primary care aspects of this care. Different healthcare professionals had different views on who should be held responsible for addressing the pharmacological treatment of osteoporosis after a hip fracture.

On many occasions immediately after the occurrence of a hip fracture, surgeons or orthopaedic surgeons are ultimately or jointly responsible for the care process within the hospital. Whilst some healthcare professionals argued that orthopaedic surgeons should be the first to start the ball rolling on osteoporosis treatment [26, 27], orthopaedic surgeons indicated that they did not consider themselves responsible for addressing pharmacological treatment of osteoporosis. As illustrated by an orthopaedic surgeon: ‘*We are busy…and so we don’t necessarily stop to discuss osteoporosis. You just kind of shrug your shoulders [when encountering osteoporosis] and go, ooh, that’s bad.*’ [26] The main reason for not feeling responsible was that orthopaedic surgeons were trained to operate and not to treat patients with medication [26]: ‘It is internal medicine’s job…to be responsible for ongoing things like osteoporosis, plus these medicines have side effects.’ [26] Another reason why orthopaedic surgeons were reluctant to take the lead in addressing osteoporosis care was that they did not want to be held responsible for the follow-up of laboratory results [26]. Whilst orthopaedic surgeons argued that other specialties, such as internal medicine or primary care, should be responsible for addressing pharmacological treatment of osteoporosis, other healthcare professionals believed that orthopaedic surgeons or Emergency Department staff should initiate the assessment and treatment [28, 29]. Some even stated that the lack of involvement of orthopaedic surgeons in the osteoporosis care process causes a significant treatment gap [26, 27]. Some specialists stated that the successful delivery of secondary fracture prevention in their centre was due to co-management of both orthopaedic surgeons and geriatricians [30].

Different viewpoints on responsibility, knowledge and capability were observed in relation to the transition from acute to primary care. Healthcare professionals working in nursing homes did not routinely consider osteoporosis management following hip fractures [31], whereas general practitioners did report addressing osteoporosis care in cases of fragility fractures in older patients [32]. Healthcare professionals at the FLS described a general willingness amongst general practitioners to address osteoporosis care; however, they noted the need for FLS involvement in the pharmacological treatment of osteoporosis after a hip fracture. They observed that general practitioners might not always have the time or capability to prioritise osteoporosis care [27].

**Table 2:** Overview of main theme 1, subthemes and illustrative quotations. Main theme 1: The *addressing* step—addressing pharmacological treatment of osteoporosis is not self-evident.

**Subtheme**	**Illustrative quotations including quotes**	**Confidence**
**I.1 Responsibility, knowledge and capability** Bennett [34], Bishop [35], Bullock [33], Cheah [30], Feldstein [26], Lerner [31], Otmar [32], Salminen [29], Verdonck [28], Wozniak [27] Specialty-specific responsibilities, competences and knowledge influence taking the initiative in screening or treating osteoporosis.	‘We are busy. . .and so we don’t necessarily stop to discuss osteoporosis. You just kind of shrug your shoulders [when encountering osteoporosis] and go, ooh, that’s bad (orthopaedic surgeon).’ [26] ‘The orthopaedic surgeons see those low-impact fractures arriving, they should say “when this is healed we perform a DXA” (general practitioner).’ [28] ‘There is this fracture and then you need to consider “why?”. Not solely because of the fall, but because she might have osteoporosis. And that’s apparently a step too far (general practitioner).’ [28] ‘I do wonder whether I know enough to explain it back to the patient (general practitioner).’ [33] ‘The thing is the GP is the best placed person to know that patient in terms of what other medications they’re on and what they can and can’t take (FLS clinician).’ [33]	High
**I.2 Identifying eligible patients** Bennet [34], Cheah [30], Drew [36], Feldstein [26], Guillemot [37], Merle [48], Wozniak [27] Patient identification depends on dedicated healthcare professionals actively trying to find eligible patients. Communication between healthcare professionals affects the identification of eligible patients.	Attendance of fracture prevention coordinators or other healthcare professionals at daily pre-operative trauma meetings was seen as effective at identifying patients at risk of further fractures. This was despite awareness that such attendance involved listening to extraneous information and relying on clinicians including discussion of all relevant patients (author’s interpretation) [36]. Relying solely on trauma meetings or ward rounds was problematic when staff were absent as it meant that there was no one to identify cases (author’s interpretation) [36]. ‘I will say (the current delivery of secondary fracture prevention) is very good. . . we have an ortho-geriatric service in the hospital which reviews all hip fractures cases. . . but I am not too sure whether other fractures are captured or not (geriatrician).’ [30] ‘Here is a typical scenario. . . an older person falls and breaks a hip. They’re admitted and get their hip fixed. . ., maybe 5 to 6 months, or maybe never, they come back to see us [general practitioner]. By that time, it [the fracture] has sort of faded into the background and we’re back to juggling all their medicines. The fracture is. . .well, nobody is really thinking about that anymore. . .and so it [osteoporosis] gets dropped off people’s radar screen (general practitioner).’ [26]	Moderate
**I.3 Perceived importance and benefit, and feasibility of treatment** Armstrong [38], Bullock [33], Cheah [30], Edwards [49], Feldstein [26], Guillemot [37], Lerner [31], Otmar [32], Verdonck [28] Uncertainties about the overall importance of osteoporosis treatment, as well as concerns about its benefits (e.g. due to old age) and feasibility (e.g. due to competing health issues or patients’ financial capacity) at the patient level, influence whether osteoporosis treatment is deemed appropriate.	‘I have some major philosophical problems with both the diagnosis and treatment of osteoporosis. . . where do you draw the line of risk and benefit?. . .the majority of people on therapies. . . will not do them good, statistically speaking (general practitioner).’ [26] ‘I have the hardest time with the very elderly who are osteoporotic and are prime candidates for treatment often they are already on lots of drugs and so there is resistance (general practitioner).’ [26] Individuals with hip fracture admitted to the medical service tend to have multiple comorbidities and thus, osteoporosis is not considered a medical priority (author’s interpretation) [49]. ‘We all come up with a different point of view as to what is actually the best way to manage this individual. Sometimes, like you say, it’s a really, really grey area, isn’t it? (FLS clinician).’ [33] ‘When the patient comes back to us, we put it more into context of what else is going on in their lives and see if it fits (general practitioner).’ [33]	Moderate

**Table 3:** Overview of main theme 2, subthemes and illustrative quotations. Main theme II: The discussing step—discussing the initiation of anti-osteoporosis medication is not a clear-cut path.

**II.1 Raising awareness** Bennett [34], Bullock [33], Cheah [30], Tahmasbi [50], Verdonck [28], Wozniak [27] Raising awareness through patient education is vital for engaging patients in discussing pharmacological treatment of osteoporosis, particularly in terms of its benefits and risks in reducing future fracture risk.	‘I don’t think they link them. That if someone broke a hip that it could possibly be due to osteoporosis (general practitioner).’ [28] ‘You need to make sure that they are first educated and aware of their options (healthcare professional working in FLS).’ [27] ‘I think that percentage of fracture risk is really powerful for the patient to start treatment and to take it. If you’re saying, “You’ve got a 15% chance of fracturing your hip. Whereas your risk of side effects from the medication is lower”, that’s powerful (FLS clinician).’ [33]	Moderate
**II.2 Being perceptive** Bennett [34], Bullock [33], Drew [36] Patients’ ability and willingness to discuss osteoporosis treatment, i.e. being perceptive, is influenced by both existing preconceptions and timing. In the acute phase patients tend to be more open to treatment to prevent new fractures. However, breaking a hip is overwhelming, so discussions should not occur too early.	‘Patients tend to, follow-up a little bit more if it hurts or if they actually think it’s important (general practitioner).’ [34] ‘You know breaking your hip is huge, there’s lots of psychological things. . . Being flexible about [the timing] is probably a bit more patient-centred (healthcare professional working in hospital).’ [36] ‘You also get quite a few patients that have already come with preconceived ideas because their friend or neighbour has already been on that medication (FLS clinician).’ [33]	Moderate
**II.3 Making sense** Bennett [34], Drew [36], Jensen [39], Jensen [40], Narayanasamy [51], Unson [52] Patients try to make sense of their fracture experience by creating a narrative in which osteoporosis treatment is considered either relevant of irrelevant. Education and ‘proof’ of underlying osteoporosis can change this reasoning to some extent.	‘Oh, just because I’d had, I’d fallen and broken my hip and I didn’t want to fall and break something else again (hip fracture patient).’ [51] ‘Mrs. Smith [one of the participants] fell a few months ago and fractured her hip. She hasn’t been taking any of “what you’re talking about—calcium or any other”. Her hip “mended just like it wasn’t even broken”’ (hip fracture patient) [52]. Suspicion and aversion of medications by a subset of patients was a common source of frustration for general practitioners. Such patients were viewed as having fixed attitudes and ‘resistance’ to advice. Education was not expected to bring about meaningful change in their knowledge or health behaviour (author’s interpretation) [34].	Moderate
**II.4 Patients-centred approach** Bullock [33], Wozniak [27] Healthcare professionals facilitate informed decision-making through a patient-centred approach. Education and flexibility, well-tailored to patients’ needs and preferences, are considered important.	‘You cannot force a patient to take a medication. That takes away their preference. You can provide the information and the supports available for the best courses of treatment (healthcare professional working in FLS).’ [27] ‘It certainly helps to provide that greater education for patients so that then they can make informed decisions; patients and families and then their wants. We are also having to be patient centred, so giving them the options with the risks and benefits and then respecting whichever choice they choose to make (healthcare professional working in FLS).’ [27] ‘You try and make it as basic as possible for some individuals and just keep the information that you give them as low key and as easy as possible (healthcare professional working in FLS).’ [33] ‘I recognise that it’s something that we could do but currently we don’t. I don’t think we find out what their values are before or what their expectations are (FLS clinician).’ [33] ‘We are still trying to ultimately preserve lives but then respect decisions of the patients themselves (healthcare professional working in FLS).’ [27]	Moderate
**II.5 Patient choice** Bullock [33], Wozniak [27] Patients decide on treatment, but their options are restricted as healthcare professionals prefer first-line treatments due to (local) guideline recommendations, costs and adherence concerns	‘From the first day they (healthcare professionals working at the FLS) realised that this could not be a cookie cutter approach. Not everyone was going to go on medication, not everyone wants to go on medication. . . (healthcare professional working in FLS).’ [27] ‘Sorry, no, they don’t have a choice [laughter] (FLS clinician).’ [33] ‘We say, “this is what we’d recommend your GP prescribes for you. That’s the treatment that would be recommended for you.” (FLS clinician).’ [33] ‘We got this directive from pharmacy and NHS England that we’ve got to prescribe the cheapest one. There is not a lot of discussion really (general practitioner).’ [33]	Moderate

A lack of knowledge was sometimes a barrier to prescribing AOMs or recognising its importance [31], or there was uncertainty about possessing adequate knowledge to properly inform patients [33]. Whilst some general practitioners felt comfortable and capable of prescribing AOMs [34], others preferred to seek guidance by discussing treatment options with specialists before prescribing [35]. Although healthcare professionals at the FLS felt well-informed about both osteoporosis and AOMs, not all believed it was their responsibility to recommend or discuss treatment with patients. They viewed this as the role of general practitioners: ‘The thing is, the GP is the best placed person to know that patient in terms of what other medications they’re on and what they can and can’t take (FLS clinician).’ [33] Moreover, in some cases, general practitioners were not permitted to prescribe AOMs, which hindered specialists’ effort to involve them in prescribing or continuing such treatment [30].

#### Subtheme I.2 identifying eligible patients

Many healthcare professionals considered identifying all patients eligible for pharmacological treatment of osteoporosis to be a delicate matter [26, 27, 36]. Within the hospital, the process of identification depended largely on professionals’ awareness and proactive search for missing cases [36]. Although some healthcare professionals considered patient identification ‘relatively easy’ and described their population as a ‘captivated audience’, they also preferred to locate patients with a hip fracture in a dedicated hospital ward, as otherwise ‘outliers’ might be missed [36]. However, some healthcare professionals highlighted the vulnerability of the translational care process and expressed their frustration of missing opportunities to address osteoporosis in a timely manner: ‘Here is a typical scenario... an older person falls and breaks a hip. They’re admitted and get their hip fixed..., maybe 5 to 6 months, or maybe never, they come back to see us [general practitioners]. By that time, it [the fracture] has sort of faded into the background and we’re back to juggling all their medicines. The fracture is...well, nobody is really thinking about that anymore...and so it [osteoporosis] gets dropped off people’s radar screen (general practitioner).’ [26] Whilst some healthcare professionals working in the hospital believed that a proper transfer from hospital (FLS) to primary care was possible by writing a clear letter to the general practitioner [27], others were more sceptical and believed that general practitioners, who were ‘already overwhelmed with their work’, were unable to process all these messages [26].

#### Subtheme I.3 perceived importance and benefit, and feasibility of treatment

Whilst some healthcare professionals emphasised the importance of addressing pharmacological treatment after the first fracture in general [30], especially in old age [37], others found themselves struggling with the importance of addressing osteoporosis care overall: ‘I have some major philosophical problems with both the diagnosis and treatment of osteoporosis... where do you draw the line of risk and benefit? ...the majority of people on therapies... will not do them good, statistically speaking (general practitioner).’ [26] Others questioned the cost-effectiveness of osteoporosis treatment after a fragility fracture in terms of public health, especially in old age [32].

Healthcare professionals described that recommending treatment of osteoporosis was often not self-evident. Whilst FLS clinicians stated that they were adept at making clinical judgements on whether treatment should be recommended, they expressed difficulties in deciding whether to recommend treatment at the patient level due to challenges in interpreting clinical guidelines [33]: ‘We all come up with a different point of view as to what is actually the best way to manage this individual. Sometimes, like you say, it’s a really, really grey area, isn’t it? (FLS clinician).’ [33] Furthermore, certain patient characteristics, such as old age and competing health issues or comorbidities, made healthcare professionals doubt the priority of addressing osteoporosis care: ‘Preferably you don’t fracture your hip, but most people with a hip prothesis are really well of, whilst a diabetic foot… you can’t add a new toe (general practitioner).’ [28] Moreover, it was described that in low-income countries, the management of osteoporosis depended on the financial ability of the patient. If the patient had insufficient financial resources to undergo a DXA scanning, osteoporosis care was sometimes not addressed [38].

### Main theme II: the discussing step—discussing the initiation of anti-osteoporosis medication is not a clear-cut path

#### Subtheme II.1 raising awareness

Healthcare professionals considered patient education a crucial first step in engaging patients in the discussion of pharmacological treatment of osteoporosis, especially since suffering a fracture does not automatically raise awareness of the possibility of underlying osteoporosis: ‘I don’t think they link them. That if someone broke a hip that it could possibly be due to osteoporosis (general practitioner).’ [28] Patient education was considered important to improve understanding of osteoporosis and its impact on one’s own health and to make patients aware of the possibility and importance of treating osteoporosis after a hip fracture [27, 34]. ‘Education is very important … increase the awareness of the risk of fractures ... what is the prevention for osteoporosis, and then what is the prevention for falls, and how to assess whether you are prone to falls or not … these are the things that we need to educate the public and at the same time educate them to seek help if there is a need (healthcare professional working in FLS).’ [30] Whilst FLS clinicians and general practitioners acknowledged the importance of communicating the benefits and risks of treatment in terms of reducing the risk of future fractures, they found it challenging to convey this information in a manner that was easily comprehensible. Furthermore, healthcare professionals did not always seem to correctly interpret the fracture risk reduction themselves [33].

#### Subtheme II.2 being perceptive

Whilst discussing osteoporosis treatment with patients, healthcare professionals considered it important to understand and address existing preconceptions of both osteoporosis and AOMs: ‘You also get quite a few patients that have already come with preconceived ideas because their friend or neighbour has already been on that medication (FLS clinician)’ [33].

Furthermore, patients and healthcare professionals felt that the timing of the conversation on osteoporosis care after a hip fracture is crucial. The impact of fracture and recovery is not to be taken lightly. Some healthcare professionals believed that the impact of fracture recovery means they have to be flexible in timing of informing the patient about osteoporosis and possible diagnosis or treatment: ‘You know breaking your hip is huge, there’s lots of psychological things… Being flexible about [the timing] is probably a bit more patient-centred (healthcare professional working in hospital).’ [36] Whilst several healthcare professionals stressed that postponing the initial talk to an outpatient setting is preferable as it gives patients a chance to recover first [26, 27], others felt that waiting too long is not good either: ‘Six months down the line, the horse has already bolted and they won’t remember what you said beforehand, so I think three months is at least the initial thing (healthcare professional working in hospital).’ [36] Some healthcare professionals preferred to assess and, if possible, start treatment in a hospital setting, as they are worried about ‘losing’ patients after discharge since they could be ‘too weak, forgot appointments or did not receive appointment letters’ [36].

#### Subtheme II.3 making sense

Many patients had difficulties in automatically perceiving a causal link between osteoporosis and their hip fracture. Dealing with this uncertainty and the invisible nature of osteoporosis poses a challenge for both patients and healthcare professionals in decision-making. Whilst some patients seemed to need ‘visual proof’ of osteoporosis before agreeing to start AOMs [36], others doubt the usefulness of doing a DXA scan in their situation: ‘Why scan me? I am more than 80 years old. Of course my bones are not as strong as when I was young. That is logical. And so what?’ [39] For some patients, the resonant experience of sustaining a hip fracture seems to influence their personal risk perception and perception of underlying osteoporosis [40]. This fracture experience motivates some patients to question their initial assumptions, making them willing to undergo diagnostic interventions or treatment of osteoporosis: ‘My bones have always been strong, so I do not think I have osteoporosis, but now I have said yes to the assessment ... you never know what age does to you now do you...I mean, how can I just stand and break my hip without having done anything?’ [40].

#### Subtheme II.4 patient-centred approach

When discussing the initiation of AOMs, healthcare professionals argue this is best done in a manner well-tailored to patients’ needs and preferences: ‘you cannot force a patient to take a medication. That takes away their preference. You can provide the information and the supports available for the best courses of treatment (FLS clinician).’ [27] Taking into account patient characteristics, such as their level of education, language proficiency, and cognitive impairments, was found to be important when providing education: ‘You try and make it as basic as possible for some individuals and just keep the information that you give them as low key and as easy as possible (FLS clinician).’ [33] Despite tailoring information provision based on patient-characteristics, healthcare professionals stated that they did not tailor information provision on the basis of values, needs or concerns, nor did they check for patients’ understanding of information [33]. Healthcare professionals felt that they could support patients in making well-informed decisions: ‘We are also having to be patient centred, so giving them the options with the risks and benefits and then respecting whichever choice they choose to make (FLS clinician)’ [27].

#### Subtheme II.5 patient choice

Whilst discussing the initiation of AOMs, patients decided whether treatment was prescribed: ‘From the first day they realized that this could not be a cookie cutter approach. Not everyone was going to go on medication, not everyone wants to go on medication…(FLS clinician).’ [27] Despite discussing the initiation of osteoporosis treatment at patient-level, healthcare professionals limited patients’ choice of AOMs by sometimes recommending only the first-line option*:* ‘Sorry, no, they don’t have a choice [laughter] (FLS clinician).’ [33] Reasons given for this limited choice included treatment preferences stated in (local) guidelines, fear of reduced medicine adherence and medicine costs: ‘We got this directive from pharmacy and NHS England that we’ve got to prescribe the cheapest one. There is not a lot of discussion really (general practitioner)’ [33].

## Discussion

The aim of this qualitative systematic review was to gain deeper insight into the experiences and preferences of older patients and healthcare professionals regarding the initiation of AOMs following a low-energy hip fracture. By exploring and accumulating the viewpoints of all stakeholders, we provided a clearer understanding of the decision-making process surrounding the initiation of AOMs after a hip fracture. We identified two steps in this process: the ‘addressing’ step and the ‘discussing’ step. The ‘addressing’ step illustrates that at the healthcare provider level, taking the initiative in treating osteoporosis depends on specialty-specific responsibilities, knowledge and capabilities, as well as the commitment to identifying eligible patients and assessing the perceived benefit and feasibility of treatment at the patient level. The ‘discussing’ step underscores the importance of patient education for raising awareness and engaging patients in osteoporosis treatment discussions. Healthcare professionals support informed decision-making through a patient-centred approach, tailored education, and flexibility. Whilst healthcare professionals set the conditions and limit treatment options by outlining the available choices, the patient ultimately decides whether or not AOMs is prescribed. The attitudes and decisions of patients and healthcare professionals determine whether AOMs are prescribed following a hip fracture in old age, thereby partly explaining the current low prescription rates.

### Comparing with literature

The lag in guideline implementation, reflected in reduced prescription rates, is a persistent phenomenon also seen in other cases of secondary prevention, such as secondary cardiovascular prevention in older adults [41–44]. Findings suggest that reduced prescribing rates for secondary cardiovascular prevention may largely be attributed to healthcare professionals consciously weighing all aspects of a situation in close dialogue with the individual patient [45]. Whether such considerations occur in the case of the initiating of AOMs after low-energy hip fracture is unknown. Consistent with our review findings, inappropriate timing of information on secondary prevention medications following ischaemic stroke or transient ischaemic attack has been perceived as a barrier to shared decision-making and treatment adherence [46].

### Clinical implications

This review has important clinical implications and proposes novel solutions beyond organisational initiatives. It demonstrates that, in addition to organisational factors, both healthcare professional-related and patient-related factors influence the decision-making process regarding the initiation of AOMs following a hip fracture in daily practice. During the initial step of addressing osteoporosis care, specialty-specific responsibilities, competencies, and knowledge determine who takes the initiative in screening for or treating osteoporosis. These finding offer valuable insights for policymakers and healthcare managers, suggesting that emphasising the importance of osteoporosis management during the training of healthcare professionals from diverse backgrounds—such as (orthopaedic) surgeons, internists, elderly care physicians, and general practitioners—may foster professional initiative in addressing osteoporosis care. Furthermore, this review highlights that healthcare professionals’ assessments of treatment benefit and feasibility at the individual patient level influence whether osteoporosis treatment is considered appropriate. This suggests that not all patients who meet guideline-based eligibility criteria are prescribed AOMs. A potential solution to this barrier may involve enhancing patient participation in the decision-making process. This could be facilitated by integrating awareness-raising and educational components into the post-hip fracture care pathway—ideally through standardised daily practices, such as including a standardised information leaflet in the discharge package.

### Strengths and limitations

As far as we know, this is the first systematic review of qualitative studies investigating the process of decision-making regarding pharmacological treatment of osteoporosis after a hip fracture in older adults. We employed a rigorous methodological approach, including the GRADE-CERQual approach to assess the confidence in our findings, the CASP checklist to evaluate methodological quality, and the ENTREQ guideline for reporting this review. However, this systematic review is not without its limitations. Firstly, some statements from healthcare professionals in this review concern a mixed population consisting of patients with a hip fracture and/or other type of fragility fracture. This may have affected the applicability of our results to the hip fracture population specifically. The fragility fracture population is a heterogeneous patient group, with the most commonly observed fracture sites being the hip, vertebrae, wrist and humerus [47]—hip fractures alone accounting for an estimated 19.6% of all fragility fractures [12]. Specific patient characteristics may influence the factors that healthcare professionals consider and address when initiating AOMs across different subgroups. However, as the aim of this systematic review was to capture all healthcare professionals’ perspectives on initiating AOMs following a hip fracture, it was considered valuable to also incorporate views relating to the broader fragility fracture population—of which individuals with hip fractures constitute a substantial subgroup—in order to provide a comprehensive overview of the available data. As some of the themes identified in studies involving the broader fragility fracture population did not differ from those observed in studies focusing exclusively on hip fracture patients, this may suggest that certain findings are transferable to other fragility fracture populations. However, further research is needed to explore healthcare professionals’ perspectives on the initiation of AOMs across different fracture populations, particularly amongst patients with hip fractures.

Furthermore, hip fracture patients showed to be underrepresented in the studies included in this systematic review. This underrepresentation may explain the lack of information on what both patients and healthcare professionals jointly consider in terms of risks and benefits during the decision-making process. We recommend that future research focusses on both groups to clarify their joint considerations.

### Conclusion

Initiating AOMs in older hip fracture patients involves a two-step decision-making process: (i) addressing treatment, and (ii) discussing treatment. These steps illustrate that, in daily practice, guideline-endorsed treatment of osteoporosis is more complex than just prescribing medication. The attitudes and decisions of patients and healthcare professionals determine whether AOMs are prescribed following a hip fracture in old age, thereby partly explaining the current low prescription rates. Beyond organisational strategies, the promotion of education and awareness concerning post-hip fracture osteoporosis care may further cultivate professional initiative and encourage active patient engagement.

## Supplementary Material

aa-25-0706-File002_afaf237
