# Supplementary material for: Initiation of anti-osteoporosis medication following hip fracture in older adults: a systematic review and thematic synthesis of qualitative studies from patient and healthcare professional perspectives
Source: Age Ageing. 2025 Aug 21;54(8):afaf237. doi: 10.1093/ageing/afaf237 (PMC12368851; doi:10.1093/ageing/afaf237)
Supplement: aa-25-0706-File002_afaf237 [file aa-25-0706-file002_afaf237.pdf]

SUPPLEMENTARY MATERIAL FOR

Initiation of anti-osteoporosis medication following hip fracture in older adults: a systematic review and thematic synthesis of qualitative studies from patient and healthcare professional perspectives

Appendix index:

Page 2-3	Appendix 1: ENTREQ Checklist (Table S1)
Page 4	Appendix 2: Detailed search methods
Page 5	Appendix 3: Critical Appraisal Skills Programme Quality Appraisal Scores (Table S2)
Page 6-11	Appendix 4: Overview of review findings and evidence (Table S3)
Page 12-23	Appendix 5: Search strings
Page 24-25	Appendix 5: References

Appendix 1: ENTREQ Checklist (Table S1)

Item	Guide and description	Reported on page
Aim	State the research question the synthesis addresses.	Page 4-5 (Introduction)
Synthesis methodology	Identify the synthesis methodology or theoretical framework which underpins the synthesis, and describe the rationale for choice of methodology (e.g. meta ethnography, thematic synthesis, critical interpretive synthesis, grounded theory synthesis, realist synthesis, meta-aggregation, meta-study, framework synthesis).	Page 7 (Methods)
Approach to searching	Indicate whether the search was pre-planned (comprehensive search strategies to seek all available studies) or iterative (to seek all available concepts until theoretical saturation is achieved).	Page 6 (Methods)
Inclusion criteria	Specify the inclusion/exclusion criteria (e.g. in terms of population, language, year limits, type of publication, study type).	Page 6 (Methods), Appendix 2 (Supplements)
Data sources	Describe the information sources used (e.g. electronic databases (MEDLINE, EMBASE, CINAHL, psychINFO, Econlit), grey literature databases (digital thesis, policy reports), relevant organisational websites, experts, information specialists, generic web searches (Google Scholar), hand searching, reference lists) and when the searches were conducted; provide the rationale for using the data sources.	Page 6 (Methods)
Electronic search strategy	Describe the literature search (e.g. provide electronic search strategies with population terms, clinical or health topic terms, experiential or social phenomena related terms, filters for qualitative research and search limits).	Page 6 (Methods), Appendix 2 and 5 (Supplements)
Study screening methods	Describe the process of study screening and sifting (e.g. title, abstract and full text review, number of independent reviewers who screened studies).	Page 6-7 (Methods), Appendix 2 (Supplements)
Study characteristics	Present the characteristics of the included studies (e.g. year of publication, country, population, number of participants, data collection, methodology, analysis, research questions).	Page 22-24, Table 1 (Results)
Study selection results	Identify the number of studies screened and provide reasons for study exclusion (e.g. for comprehensive searching, provide numbers of studies screened and reasons for exclusion indicated in a figure/flowchart; for iterative searching describe reasons for study exclusion and inclusion based on modifications the research question and/or contribution to theory development).	Page 29, Figure 1 (Results)
Rationale for appraisal	Describe the rationale and approach used to appraise the included studies or selected findings (e.g. assessment of conduct (validity and robustness), assessment of reporting (transparency), assessment of content and utility of the findings).	Page 7 (Methods), Page 9 (Results), Appendix 3 and 4 (Supplements)
Appraisal items	State the tools, frameworks and criteria used to appraise the studies or selected findings (e.g. Existing tools: CASP, QARI, COREQ, Mays and Pope [25]; reviewer developed tools; describe the domains assessed: research team, study design, data analysis and interpretations, reporting).	Page 7 (Methods), Page 9 (Results)

Appraisal process	Indicate whether the appraisal was conducted independently by more than one reviewer and if consensus was required.	Page 7 (Methods)
Appraisal results	Present results of the quality assessment and indicate which articles, if any, were weighted/excluded based on the assessment and give the rationale.	Page 9 (Results), Appendix 3 (Supplements)
Data extraction	Indicate which sections of the primary studies were analysed and how were the data extracted from the primary studies? (e.g. all text under the headings "results /conclusions" were extracted electronically and entered into a computer software).	Page 7 (Methods)
Software	State the computer software used, if any.	Page 7 (Methods)
Number of reviewers	Identify who was involved in coding and analysis.	Page 7 (Methods)
Coding	Describe the process for coding of data (e.g. line by line coding to search for concepts).	Page 7 (Methods)
Study comparison	Describe how were comparisons made within and across studies (e.g. subsequent studies were coded into pre-existing concepts, and new concepts were created when deemed necessary).	Page 7 (Methods)
Derivation of themes	Explain whether the process of deriving the themes or constructs was inductive or deductive.	Page 7 (Methods)
Quotations	Provide quotations from the primary studies to illustrate themes/constructs, and identify whether the quotations were participant quotations or the author's interpretation.	Page 10-14 (Results) and Page 25-28, Table 2 and 3 (Results)
Synthesis output	Present rich, compelling and useful results that go beyond a summary of the primary studies (e.g. new interpretation, models of evidence, conceptual models, analytical framework, development of a new theory or construct).	Page 10-14 (Results), Page 15-17 (Discussion and conclusion and implications)

Appendix 2: Detailed search methods (including Figure S1)

A systematic search was undertaken on 31-07-2023, which yielded 1,334 results after removal of duplicates. An updated search was conducted on 07-02-2025, yielding an additional 142 results. The following inclusion and exclusion criteria were used:

Inclusion criteria:
1) All types of written qualitative studies involving healthcare professionals providing care for, or patients with, a hip fracture.
2) Data were collected through interviews, focus groups, or observations.
3) No publication date constraints.
4) For patient data the focus is on hip fracture, hence studies with a mixed population were data on hip fractures were not identifiable were excluded.
5) For healthcare professionals the focus is on hip fracture patients or fragility fracture patients, hence studies with a mixed population were data on hip or fragility fracture patients were not identifiable were excluded.

Exclusion criteria:
1) Patients with secondary osteoporosis were excluded.
2) Non-English articles were excluded to prevent cultural and linguistic bias in translation.

Study selection and process of exclusion

Studies were selected in a systematic manner by two authors independently based on title, abstract and full-text using the predefined inclusion and exclusion criteria. Articles were excluded using the following labels:
1) Irrelevant topic; meaning that the study focused on a completely different topic or research field (e.g. basic medical research).
2) No abstract or full-text; meaning that no abstract or full-text could be retrieved.
3) Duplicate; meaning that the study was a duplicate that was missed during the process of initial removal of duplicates.|
4) Not qualitative; meaning that the study was using a methodology that was not qualitative (e.g. Delphi study).
5) Not directly linked to hip fracture patients; meaning that the study did not contain data on identifiable hip fracture patients in case of patient data, or identifiable hip fracture patients or fragility fracture patients in case of data on healthcare professionals; e.g. quotations could not be directly linked to these populations.
6) No post-fracture osteoporosis management; meaning that the study did not contain data on post-fracture osteoporosis management.
7) No extractable data; meaning that there was no data eligible for extraction.
8) No original data; meaning that the data of the study was accumulated from other studies, e.g. a systematic review or meta-analysis.

Appendix 3: Critical Appraisal Skills Programme Quality Appraisal Scores (Table S2)

	Q1	Q2	Q3	Q4	Q5	Q6	Q7	Q8	Q9	Q10
**Article**										
Armstrong E 2022	Yes	Yes	Yes	Yes	No	Can’t tell	Yes	No	Yes	Yes
Bennett MJ 2023	Yes	Yes	Can't tell	Can’t tell	Yes	No	Yes	Yes	Yes	Yes
Bishop S 2023	Yes	Yes	Yes	Yes	Can't tell	Can't tell	Yes	Yes	Yes	Yes
Bullock L 2024	Yes	Yes	Can’t tell	Yes	Yes	Can't tell	Yes	Yes	Yes	Yes
Cheah MH 2024	Yes	Yes	Yes	Yes	Yes	Can’t tell	Yes	Yes	Yes	Yes
Drew S 2016	Yes	Yes	Yes	Yes	Yes	No	Yes	Can’t tell	No	Yes
Edwards BJ 2012	No	Yes	Can't tell	Can't tell	Yes	Can't tell	Yes	No	No	Yes
Feldstein AC 2008	Yes	Yes	Can’t tell	Yes	Yes	No	Yes	Can’t tell	Yes	Yes
Guillemot JR 2024	Yes	Yes	Yes	Yes	Yes	Can’t tell	Yes	Yes	Can’t tell	Yes
Jensen CM 2017	Yes	Yes	Can’t tell	No	Yes	No	Yes	Yes	Yes	Yes
Jensen CM 2019	Yes	Yes	Yes	No	Yes	No	Yes	Yes	Yes	Yes
Lerner ET 2025	Yes	Yes	Can’t tell	Yes	Yes	Can’t tell	Can’t tell	Can’t tell	Can’t tell	Yes
Merle B 2019	Yes	Yes	Yes	Yes	Yes	Can't tell	Yes	Yes	Yes	Yes
Otmar R 2012	Yes	Yes	Yes	Yes	Yes	Can’t tell	Yes	Yes	Yes	Yes
Naryanasamy M 2022	Yes	Yes	Yes	No	Yes	No	Yes	Yes	Yes	Yes
Salminen H 2019	Yes	Yes	Can’t tell	Yes	Yes	Can’t tell	Yes	Yes	Yes	Yes
Tahmasbi F 2024	Yes	Yes	Can’t tell	Yes	Yes	Can’t tell	Yes	Yes	Yes	Yes
Unson CG 2003	Yes	Yes	No	Yes	Yes	No	No	Yes	Yes	Yes
Verdonck C 2023	Yes	Yes	Can’t tell	Can’t tell	Yes	Can’t tell	Yes	Yes	Yes	Yes
Wozniak LA 2020	Yes	Yes	Yes	Yes	Yes	No	Yes	Yes	Yes	Yes

Q1: Was there a clear statement of the aims of research?, Q2: Is qualitative methodology appropriate?, Q3: Was the research design appropriate to address the aims of the research?, Q4: Was the recruitment strategy appropriate to address the aims of the research?, Q5: Was the data collected in a way that addressed the research issue?, Q6: Has the relationship between researcher and participant been adequately considered?, Q7: Have ethical issues been taken into consideration?, Q8: Was the data analysis sufficiently rigorous?, Q9: Is there a clear statement of findings?, Q10: How valuable is the research?

Appendix 4: **CERQual Evidence Profile and Summary of Qualitative Findings (SoQF) table** **(Table S3)**

**Summary of review finding**	**Studies contributing to the review finding**	**Methodological limitations**	**Coherence**	**Adequacy**	**Relevance**	**CERQual assessment of confidence in the evidence**	**Explanation of CERQual assessment**
**Main theme 1: The *addressing* step – addressing pharmacological treatment of osteoporosis is not self-evident**							
**I.1 Responsibility, knowledge and capability**<br><br>Specialty-specific responsibilities, competences and knowledge influence taking the initiative in screening or treating osteoporosis.	Bennett[1], Bishop[2], Bullock[3], Cheah[4], Feldstein[5], Lerner[6], Otmar[7], Salminen[8], Verdonck[9], Wozniak[10]	Minor concerns (Author reflexivity was not considered in 10 studies, and 6 studies lacked rationale for the study design. Additionally, 2 studies lacked detailed information on recruitment and data analysis, and information was missing on data collection (1 study), ethical issues (1 study), and a clear statement of findings (1 study)).	No or very minor concerns (ten studies contributing to this finding; the finding reflects the variety of different viewpoints expressed by healthcare professionals in the primary studies).	Minor concerns (a sufficient quantity of rich data with different perspectives from 6 studies contributed to the finding, 3 studies lacked richness in data).	Minor-moderate concerns, five studies focused on post-fracture population, four on osteoporosis population, all studies had partial relevance due to dissimilar study aims compared to the review.	High confidence.	Minor methodological limitations, no-minor concerns regarding the coherence of data and, minor concerns with data adequacy.
**I.2 Identifying eligible patients**<br><br>Patient	Bennet[1], Cheah[4], Drew[11], Feldstein[5], Guillemot[12],	Minor concerns (Author reflexivity was not considered in 8 studies, and 3	Moderate concerns (eight studies contributing to	Minor concerns (sufficient amount of	Minor-moderate concerns, one study focused on hip fracture	Moderate confidence.	Minor methodological limitations, moderate

identification depends on dedicated healthcare professionals actively trying to find eligible patients. Communication between healthcare providers affects the identification of eligible patients.	Merle[13], Tahamasbi[14], Wozniak[10]	studies lacked rationale for the study design. Additionally, 2 studies lacked detailed information on data analysis, 2 studies lacked a clear statement of findings, and information was lacking on recruitment strategy for 1 study).	this finding; four studies described the impact of (lack of) a dedicated healthcare professional in patient identification, in relation to communication between healthcare professionals three studies were expressing problems with communication between professionals, one contradicting it based on experience of the interviewed professionals.	rich data from different healthcare professionals).	population, five studies on post-fracture population, two on osteoporosis population, all studies had partial relevance due to dissimilar study aims compared to the review.		concerns regarding the coherence of data and, minor concerns with data adequacy.
**I.3 Perceived importance and benefit, and feasibility of treatment**<br><br>Uncertainties about the overall importance of	Armstrong[15], Bullock[3], Cheah[4], Edwards[16], Feldstein[5], Guillemot[12], Lerner[6], Otmar[7], Verdonck[9]	Minor-Moderate Concerns: (Author reflexivity was not considered in 9 studies, and 2 studies lacked rationale for the study design. Additionally, 3 studies lacked	Minor-moderate concerns (nine studies contributing to finding; uncertainty mentioned in three studies, feasibility	Minor concerns (sufficient amount of rich data from different healthcare professionals).	Minor-moderate concerns, one study focuses on hip-fracture population, four studies on post-fracture population, four on osteoporosis	Moderate confidence.	Minor-moderate methodological limitations, minor-moderate concerns regarding the coherence of data and, minor

osteoporosis treatment, as well as concerns about its benefits (e.g. due to old age) and feasibility (e.g. due to competing health issues or patients' financial capacity) at the patient level, influence whether osteoporosis treatment is deemed appropriate.		detailed information on data analysis, 2 studies lacked a clear statement of findings, and information was lacking on ethical issues in 1 study).	mentioned in two studies, costs mentioned in two studies with contradictory findings, probably related to high/low-income setting; competing health issues mentioned in three studies, similar findings; old age mentioned in one study, other factors in one study).		population, all studies had partial relevance due to dissimilar study aims compared to the review.		concerns with data adequacy.
**Main theme II: The *discussing* step - discussing the initiation of anti-osteoporosis medication is not a clear-cut path**							
**II.1 Raising awareness** Raising awareness through patient education is vital for engaging patients in discussing pharmacological treatment of osteoporosis, particularly in terms of its benefits and	Bennett[1], Bullock[3], Cheah[4], Tahmasbi[14], Verdonck[9], Wozniak[10]	Minor-Moderate Concerns: (Author reflexivity was not considered in 6 studies, and 4 studies lacked rationale for the study design. Additionally, 2 studies lacked detailed information on recruitment strategy).	Minor to moderate concerns (six studies supporting finding; stressing importance of education, 1 study mentioned risk communication).	Minor concerns (sufficient amount of rich data from different healthcare professionals).	Minor-moderate concerns, five studies focused on post-fracture population, one study on osteoporosis population, all studies had partial relevance due to dissimilar study aims compared to the review.	Moderate confidence.	Minor-moderate methodological limitations, minor-moderate concerns regarding the coherence of data and, minor-moderate concerns with data adequacy.

risks in reducing future fracture risk							
**I.2 Being perceptive**<br><br>Patients' ability and willingness to discuss osteoporosis treatment, i.e. being perceptive, is influenced by both existing preconceptions and timing. In the acute phase patients tend to be more open to treatment to prevent new fractures. However, breaking a hip is overwhelming, so discussions should not occur too early.	Bennett[1], Bullock[3], Drew[11]	Moderate Concerns: (Author reflexivity was not considered in 3 studies, and 2 studies lacked rationale for the study design. Additionally, 1 study lacked detailed information on the recruitment strategy, and information was lacking on ethical issues in 1 study and data analysis in 1 study).	Minor to moderate concerns (three studies show similar aspects of finding; two studies on patient perceptiveness, two studies in relation to timing, and two studies indicating that flexible timing is important, one study highlighted the importance of addressing preconceptions, as they affect the perception of osteoporosis medication).	Minor concerns (sufficient amount of rich data from different patients and healthcare professionals).	Minor-moderate concerns, one study focuses on hip-fracture population, two on post-fracture population, all studies had partial relevance due to dissimilar study aims compared to the review.	Moderate confidence.	Moderate methodological limitations, minor-moderate concerns regarding the coherence of data and, minor-moderate concerns with data adequacy.
**II.3 Making sense**<br><br>Patients try to make sense of their fracture experience by creating a narrative in which osteoporosis	Bennett[1], Drew[11], Jensen[17], Jensen[18], Narayanasamy[19], Unson[20]	Moderate concerns (Author reflexivity was not considered in 6 studies, and 3 studies lacked rationale for the study design. Additionally, 4	Minor-moderate concerns (six studies supporting finding; effect of fracture experience discussed in four	Minor-moderate concerns (sufficient amount of data from different patients,	Minor-moderate concerns, three studies focused on hip fracture patients, two studies on osteoporosis patients, one on	Moderate confidence.	Moderate methodological limitations, minor-moderate concerns regarding the coherence of data and, minor-

treatment is considered either relevant of irrelevant. Education and 'proof' of underlying osteoporosis can change this reasoning to some extent.		studies lacked detailed information on the recruitment strategy, and information was lacking on ethical issues in 1 study, data analysis in 1 study, and a clear statement of findings in 1 study).	studies, with two studies indicating that fracture experience motivated patients to start treatment, two other studies contained contradictory data, the narrative patients used was described in four studies, one study indicated the limited effect of patient education, one the need for proof).	limited amount of data from healthcare professionals).	post-fracture patients, all studies had partial relevance due to dissimilar study aim compared to the review).		moderate concerns with data adequacy.
**II.4 Patient-centred approach**<br><br>Healthcare professionals facilitate informed decision-making through a patient-centred approach. Education and flexibility, well-tailored to patients' needs and	Bullock[3], Wozniak[10]	No-Minor Concerns: (Author reflexivity was not considered in 2 studies, and 1 study lacked rationale for the study design).	Moderate concerns (two studies contributed to this review finding: one advocating person-centred care, and both emphasizing the importance of flexibility and	Moderate concerns (two studies contributing contains data from different perspectives).	Minor-moderate (two studies focusing on post-fracture population, both studies had partial relevance due to dissimilar study aim compared to the review).	Moderate confidence.	No-minor methodological limitations, moderate concerns regarding the coherence of data and, minor-moderate concerns with data adequacy.

preferences, are considered important.			well-tailored care).				
**II.5 Patient choice**<br><br>Patients decide on treatment, but their options are restricted as healthcare professionals prefer first-line treatments due to (local) guideline recommendations, costs and adherence concerns.	Bullock[3], Wozniak[10]	No-Minor Concerns: (Author reflexivity was not considered in 2 studies, and 1 study lacked rationale for the study design).	Moderate concerns (two studies contributed to this review finding, both indicating that the patient makes the final decision. One study highlighted that healthcare professionals offer a restricted choice to patients regarding medication).	Moderate concerns (two studies contributing contains data from different perspectives).	Minor-moderate (two studies focusing on post-fracture population, both studies had partial relevance due to dissimilar study aim compared to the review).	Moderate confidence.	No-minor methodological limitations, moderate concerns regarding the coherence of data and, moderate concerns with data adequacy.

Appendix 5: Search strings

Search string for Pubmed:
((("osteoporotic hip fracture"[tw] OR "osteoporotic hip fractures"[tw] OR "osteoporosis hip fracture"[tw] OR "hip osteoporotic fracture"[tw] OR "hip osteoporotic fractures"[tw] OR (("osteoporotic"[tw] AND "hip"[tw] AND "fracture"[tw]) OR ("osteoporotic"[tw] AND "hip"[tw] AND "fractures"[tw]) OR ("osteoporotic"[tw] AND "hip"[tw] AND "fractured"[tw]) OR ("osteoporotic"[tw] AND "hip"[tw] AND "fracturing"[tw]) OR ("osteoporosis"[tw] AND "hip"[tw] AND "fracture"[tw]) OR ("osteoporosis"[tw] AND "hip"[tw] AND "fractures"[tw]) OR ("osteoporosis"[tw] AND "hip"[tw] AND "fractured"[tw]) OR ("osteoporosis"[tw] AND "hip"[tw] AND "fracturing"[tw])) OR ("Hip Fractures"[mesh] AND "Osteoporotic Fractures"[mesh]) OR ("Hip Fractures"[mesh] AND "Osteoporosis"[mesh]) OR (("Hip Fractures"[mesh] OR "Hip Fractures"[tw] OR "Femoral Neck Fractures"[tw] OR "Proximal Femoral Fractures"[tw] OR "Intertrochanteric Fractures"[tw] OR "Trochanteric Fractures"[tw] OR "Subtrochanteric Fractures"[tw] OR "Pertrochanteric fractures"[tw] OR "Hip Fracture"[tw] OR "Femoral Neck Fracture"[tw] OR "Proximal Femoral Fracture"[tw] OR "Intertrochanteric Fracture"[tw] OR "Trochanteric Fracture"[tw] OR "Subtrochanteric Fracture"[tw] OR "Pertrochanteric fracture"[tw] OR (("Hip"[tw] OR "Hips"[tw]) AND ("Fracture"[tw] OR "Fractures"[tw] OR "fractur*"[tw]))) AND ("Osteoporosis"[mesh] OR "Osteoporosis"[tw] OR "Osteoporotic"[tw] OR "Osteoporo*"[tw] OR "Osteoporotic Fractures"[mesh])) OR "Fragility fracture"[tw] OR "Fragility fractures"[tw] OR "Frailty fracture "[tw] OR "Frailty fractures"[tw] OR "Osteoporosis Liaison"[tw] OR (("Fragility"[tw] AND "fracture"[tw]) OR ("Fragility"[tw] AND "fractures"[tw]) OR ("Frailty"[tw] AND "fracture"[tw]) OR ("Frailty"[tw] AND "fractures"[tw]) OR ("Fracture"[tw] AND "Liaison"[tw]) OR ("Osteoporosis"[tw] AND "Liaison"[tw]) OR ("low"[tw] AND "trauma"[tw] AND "fracture"[tw]) OR ("low"[tw] AND "trauma"[tw] AND "fractures"[tw])) OR "Fracture Liaison Service"[tw] OR "Fracture Liaison"[tw] OR "low trauma fracture"[tw] OR "low trauma fractures"[tw]) AND ("Qualitative Research"[Mesh] OR "Qualitative Research"[tw] OR "qualitative studies"[tw] OR "qualitative study"[tw] OR "qualitative analysis"[tw] OR "qualitative"[tw] OR "qualitatively"[tw] OR "qualitativ*"[tw] OR "Focus Groups"[Mesh] OR "Focus Groups"[tw] OR "Focus Group"[tw] OR "Anthropology, Cultural"[Mesh] OR "Cultural Anthropology"[tw] OR "Material Culture"[tw] OR "Ethnography"[tw] OR "Interviews as Topic"[Mesh] OR "Interview, Psychological"[Mesh] OR "Interviews"[tw] OR "Interview"[tw] OR "Interviewed"[tw] OR "Interviewing"[tw] OR "Interviewer"[tw] OR "Interviewers"[tw] OR "Interview*"[tw] OR "Empirical Research"[Mesh] OR "Empirical Research"[tw] OR "direct observation"[tw] OR "direct observations"[tw] OR "direct observation*"[tw] OR "Delphi Technique"[Mesh] OR "Delphi Technique"[tw] OR "Delphi Method"[tw] OR "Delphi Methods"[tw] OR "Delphi Studies"[tw] OR "Delphi study"[tw] OR "Delphi Technic"[tw] OR "Delphi Techniques"[tw] OR "Delphi"[tw] OR "RAND UCLA appropriateness"[tw] OR "RAND appropriateness"[tw] OR "UCLA appropriateness"[tw]) AND english[la]) OR (("Fractures, Bone"[majr] OR "Fracture"[ti] OR "Fractures"[tiab] OR "fractur*"[tiab]) AND ("Osteoporosis"[mesh] OR "Osteoporosis"[tw] OR "Osteoporotic"[tw] OR "Osteoporo*"[tw] OR "Osteoporotic Fractures"[mesh]) AND ("Qualitative Research"[Mesh] OR "Qualitative Research"[tw] OR "qualitative studies"[tw] OR "qualitative study"[tw] OR "qualitative analysis"[tw] OR "qualitative"[ti] OR "qualitatively"[ti] OR "qualitativ*"[ti] OR "Focus Groups"[Mesh] OR "Focus Groups"[tw] OR "Focus Group"[tw] OR "Anthropology, Cultural"[Mesh] OR "Cultural Anthropology"[tw] OR "Material Culture"[tw] OR "Ethnography"[tw] OR "Interviews as Topic"[Mesh] OR "Interview, Psychological"[Mesh] OR "Interviews"[tw] OR "Interview"[tw] OR "Interviewed"[tw] OR "Interviewing"[tw] OR "Interviewer"[tw] OR "Interviewers"[tw] OR "Interview*"[tw] OR "Empirical Research"[Mesh] OR "Empirical Research"[tw] OR "direct observation"[tw] OR "direct observations"[tw] OR "direct observation*"[tw] OR "Delphi Technique"[Mesh] OR "Delphi Technique"[tw] OR "Delphi Method"[tw] OR "Delphi Methods"[tw] OR "Delphi Studies"[tw] OR "Delphi study"[tw] OR "Delphi Technic"[tw] OR "Delphi Techniques"[tw] OR "Delphi"[tw] OR "RAND UCLA appropriateness"[tw] OR "RAND appropriateness"[tw] OR "UCLA appropriateness"[tw]) AND english[la]))

Search string for Embase:
((("osteoporotic hip fracture".ti,ab OR "osteoporotic hip fractures".ti,ab OR "osteoporosis hip fracture".ti,ab OR "hip osteoporotic fracture".ti,ab OR "hip osteoporotic fractures".ti,ab OR (("osteoporotic" AND "hip" AND "fracture") OR ("osteoporotic" AND "hip" AND "fractures") OR ("osteoporotic" AND "hip" AND "fractured") OR ("osteoporotic" AND "hip" AND "fracturing") OR ("osteoporosis" AND "hip" AND "fracture") OR ("osteoporosis" AND "hip" AND "fractures") OR ("osteoporosis" AND "hip" AND "fractured") OR ("osteoporosis" AND "hip" AND "fracturing")).ti,ab OR (exp *"Hip Fracture"/ AND exp *"Fragility Fracture"/) OR (exp *"Hip Fractures"/ AND exp *"Osteoporosis"/) OR ((exp *"Hip Fracture"/ OR "Hip Fractures".ti,ab OR "Femoral Neck Fractures".ti,ab OR "Proximal Femoral Fractures".ti,ab OR "Intertrochanteric Fractures".ti,ab OR "Trochanteric Fractures".ti,ab OR "Subtrochanteric Fractures".ti,ab OR "Pertrochanteric fractures".ti,ab OR "Hip Fracture".ti,ab OR "Femoral Neck Fracture".ti,ab OR "Proximal Femoral Fracture".ti,ab OR "Intertrochanteric Fracture".ti,ab OR "Trochanteric Fracture".ti,ab OR "Subtrochanteric Fracture".ti,ab OR "Pertrochanteric fracture".ti,ab OR (("Hip".ti,ab OR "Hips".ti,ab) AND ("Fracture".ti,ab OR "Fractures".ti,ab OR "fractur*".ti,ab))) AND (exp *"Osteoporosis"/ OR "Osteoporosis".ti,ab OR "Osteoporotic".ti,ab OR "Osteoporo*".ti,ab OR exp *"Fragility Fracture"/)) OR exp *"Fragility Fracture"/ OR "Fragility fracture".ti,ab OR "Fragility fractures".ti,ab OR "Frailty fracture ".ti,ab OR "Frailty fractures".ti,ab OR (("Fragility" AND "fracture") OR ("Fragility" AND "fractures") OR ("Frailty" AND "fracture") OR ("Frailty" AND "fractures")).ti,ab OR "Fracture Liaison Service".ti,ab OR "Fracture Liaison".ti,ab OR "Osteoporosis Liaison".ti,ab OR "low trauma fracture".ti,ab OR "low trauma fractures".ti,ab OR (("Osteoporosis" AND "Liaison") OR ("Fracture" AND "Liaison") OR ("low" AND "trauma" AND "fracture") OR ("low" AND "trauma" AND "fractures")).ti,ab) AND (exp *"Qualitative Research"/ OR "Qualitative Research".ti,ab OR "qualitative studies".ti,ab OR "qualitative study".ti,ab OR "qualitative analysis".ti,ab OR "qualitative".ti,ab OR "qualitatively".ti,ab OR "qualitativ*".ti,ab OR "Focus Groups".ti,ab OR "Focus Group".ti,ab OR exp *"Cultural Anthropology"/ OR "Cultural Anthropology".ti,ab OR "Material Culture".ti,ab OR "Ethnography".ti,ab OR exp *"Interview"/ OR "Interviews".ti,ab OR "Interview".ti,ab OR "Interviewed".ti,ab OR "Interviewing".ti,ab OR "Interviewer".ti,ab OR "Interviewers".ti,ab OR "Interview*".ti,ab OR exp *"Empirical Research"/ OR "Empirical Research".ti,ab OR "direct observation".ti,ab OR "direct observations".ti,ab OR "direct observation*".ti,ab OR exp *"Delphi Study"/ OR "Delphi Technique".ti,ab OR "Delphi Method".ti,ab OR "Delphi Methods".ti,ab OR "Delphi Studies".ti,ab OR "Delphi study".ti,ab OR "Delphi Technic".ti,ab OR "Delphi Techniques".ti,ab OR "Delphi".ti,ab OR "RAND UCLA appropriateness".ti,ab OR "RAND appropriateness".ti,ab OR "UCLA appropriateness".ti,ab) AND english.la) OR ((exp *"Fracture"/ OR "Fracture".ti OR "Fractures".ti OR "fractur*".ti) AND (exp *"Osteoporosis"/ OR "Osteoporosis".ti,ab OR "Osteoporotic".ti,ab OR "Osteoporo*".ti,ab OR exp *"Fragility Fracture"/) AND (exp *"Qualitative Research"/ OR "Qualitative Research".ti,ab OR "qualitative studies".ti,ab OR "qualitative study".ti,ab OR "qualitative analysis".ti,ab OR "qualitative".ti OR "qualitatively".ti OR "qualitativ*".ti OR "Focus Groups".ti,ab OR "Focus Group".ti,ab OR exp *"Cultural Anthropology"/ OR "Cultural Anthropology".ti,ab OR "Material Culture".ti,ab OR "Ethnography".ti,ab OR exp *"Interview"/ OR "Interviews".ti,ab OR "Interview".ti,ab OR "Interviewed".ti,ab OR "Interviewing".ti,ab OR "Interviewer".ti,ab OR "Interviewers".ti,ab OR "Interview*".ti,ab OR exp *"Empirical Research"/ OR "Empirical Research".ti,ab OR "direct observation".ti,ab OR "direct observations".ti,ab OR "direct observation*".ti,ab OR exp *"Delphi Study"/ OR "Delphi Technique".ti,ab OR "Delphi Method".ti,ab OR "Delphi Methods".ti,ab OR "Delphi Studies".ti,ab OR "Delphi study".ti,ab OR "Delphi Technic".ti,ab OR "Delphi Techniques".ti,ab OR "Delphi".ti,ab OR "RAND UCLA appropriateness".ti,ab OR "RAND appropriateness".ti,ab OR "UCLA appropriateness".ti,ab) AND english.la))

- NOT conference review.pt
- NOT (conference review or conference abstract).pt
- AND (conference abstract).pt

Search string for Web of Science:

(((TI=("osteoporotic hip fracture" OR "osteoporotic hip fractures" OR "osteoporosis hip fracture" OR "hip osteoporotic fracture" OR "hip osteoporotic fractures" OR ("osteoporotic" AND "hip" AND "fracture") OR ("osteoporotic" AND "hip" AND "fractures") OR ("osteoporotic" AND "hip" AND "fractured") OR ("osteoporotic" AND "hip" AND "fracturing") OR ("osteoporosis" AND "hip" AND "fracture") OR ("osteoporosis" AND "hip" AND "fractures") OR ("osteoporosis" AND "hip" AND "fractured") OR ("osteoporosis" AND "hip" AND "fracturing") OR (("Hip Fracture" OR "Hip Fractures" OR "Femoral Neck Fractures" OR "Proximal Femoral Fractures" OR "Intertrochanteric Fractures" OR "Trochanteric Fractures" OR "Subtrochanteric Fractures" OR "Pertrochanteric fractures" OR "Hip Fracture" OR "Femoral Neck Fracture" OR "Proximal Femoral Fracture" OR "Intertrochanteric Fracture" OR "Trochanteric Fracture" OR "Subtrochanteric Fracture" OR "Pertrochanteric fracture" OR (("Hip" OR "Hips") AND ("Fracture" OR "Fractures" OR "fractur*"))) AND ("Osteoporosis" OR "Osteoporosis" OR "Osteoporotic" OR "Osteoporo*" OR "Fragility Fracture")) OR "Fragility Fracture" OR "Fragility fracture" OR "Fragility fractures" OR "Frailty fracture " OR "Frailty fractures" OR ("Fragility" AND "fracture") OR ("Fragility" AND "fractures") OR ("Frailty" AND "fracture") OR ("Frailty" AND "fractures") OR "Fracture Liaison Service" OR "Fracture Liaison" OR "Osteoporosis Liaison" OR "low trauma fracture" OR "low trauma fractures" OR ("Osteoporosis" AND "Liaison") OR ("Fracture" AND "Liaison") OR ("low" AND "trauma" AND "fracture") OR ("low" AND "trauma" AND "fractures")) OR AK=("osteoporotic hip fracture" OR "osteoporotic hip fractures" OR "osteoporosis hip fracture" OR "hip osteoporotic fracture" OR "hip osteoporotic fractures" OR ("osteoporotic" AND "hip" AND "fracture") OR ("osteoporotic" AND "hip" AND "fractures") OR ("osteoporotic" AND "hip" AND "fractured") OR ("osteoporotic" AND "hip" AND "fracturing") OR ("osteoporosis" AND "hip" AND "fracture") OR ("osteoporosis" AND "hip" AND "fractures") OR ("osteoporosis" AND "hip" AND "fractured") OR ("osteoporosis" AND "hip" AND "fracturing") OR (("Hip Fracture" OR "Hip Fractures" OR "Femoral Neck Fractures" OR "Proximal Femoral Fractures" OR "Intertrochanteric Fractures" OR "Trochanteric Fractures" OR "Subtrochanteric Fractures" OR "Pertrochanteric fractures" OR "Hip Fracture" OR "Femoral Neck Fracture" OR "Proximal Femoral Fracture" OR "Intertrochanteric Fracture" OR "Trochanteric Fracture" OR "Subtrochanteric Fracture" OR "Pertrochanteric fracture" OR (("Hip" OR "Hips") AND ("Fracture" OR "Fractures" OR "fractur*"))) AND ("Osteoporosis" OR "Osteoporosis" OR "Osteoporotic" OR "Osteoporo*" OR "Fragility Fracture")) OR "Fragility Fracture" OR "Fragility fracture" OR "Fragility fractures" OR "Frailty fracture " OR "Frailty fractures" OR ("Fragility" AND "fracture") OR ("Fragility" AND "fractures") OR ("Frailty" AND "fracture") OR ("Frailty" AND "fractures") OR "Fracture Liaison Service" OR "Fracture Liaison" OR "Osteoporosis Liaison" OR "low trauma fracture" OR "low trauma fractures" OR ("Osteoporosis" AND "Liaison") OR ("Fracture" AND "Liaison") OR ("low" AND "trauma" AND "fracture") OR ("low" AND "trauma" AND "fractures")) OR AB=("osteoporotic hip fracture" OR "osteoporotic hip fractures" OR "osteoporosis hip fracture" OR "hip osteoporotic fracture" OR "hip osteoporotic fractures" OR ("osteoporotic" AND "hip" AND "fracture") OR ("osteoporotic" AND "hip" AND "fractures") OR ("osteoporotic" AND "hip" AND "fractured") OR ("osteoporotic" AND "hip" AND "fracturing") OR ("osteoporosis" AND "hip" AND "fracture") OR ("osteoporosis" AND "hip" AND "fractures") OR ("osteoporosis" AND "hip" AND "fractured") OR ("osteoporosis" AND "hip" AND "fracturing") OR (("Hip Fracture" OR "Hip Fractures" OR "Femoral Neck Fractures" OR "Proximal Femoral Fractures" OR "Intertrochanteric Fractures" OR "Trochanteric Fractures" OR "Subtrochanteric Fractures" OR "Pertrochanteric fractures" OR "Hip Fracture" OR "Femoral Neck Fracture" OR

"Proximal Femoral Fracture" OR "Intertrochanteric Fracture" OR "Trochanteric Fracture" OR "Subtrochanteric Fracture" OR "Pertrochanteric fracture" OR (("Hip" OR "Hips") AND ("Fracture" OR "Fractures" OR "fractur*"))) AND ("Osteoporosis" OR "Osteoporosis" OR "Osteoporotic" OR "Osteoporo*" OR "Fragility Fracture")) OR "Fragility Fracture" OR "Fragility fracture" OR "Fragility fractures" OR "Frailty fracture " OR "Frailty fractures" OR ("Fragility" AND "fracture") OR ("Fragility" AND "fractures") OR ("Frailty" AND "fracture") OR ("Frailty" AND "fractures") OR "Fracture Liaison Service" OR "Fracture Liaison" OR "Osteoporosis Liaison" OR "low trauma fracture" OR "low trauma fractures" OR ("Osteoporosis" AND "Liaison") OR ("Fracture" AND "Liaison") OR ("low" AND "trauma" AND "fracture") OR ("low" AND "trauma" AND "fractures"))) AND (TI=("Qualitative Research" OR "Qualitative Research" OR "qualitative studies" OR "qualitative study" OR "qualitative analysis" OR "qualitative" OR "qualitatively" OR "qualitativ*" OR "Focus Groups" OR "Focus Group" OR "Cultural Anthropology" OR "Cultural Anthropology" OR "Material Culture" OR "Ethnography" OR "Interview" OR "Interviews" OR "Interview" OR "Interviewed" OR "Interviewing" OR "Interviewer" OR "Interviewers" OR "Interview*" OR "Empirical Research" OR "Empirical Research" OR "direct observation" OR "direct observations" OR "direct observation*" OR "Delphi Study" OR "Delphi Technique" OR "Delphi Method" OR "Delphi Methods" OR "Delphi Studies" OR "Delphi study" OR "Delphi Technic" OR "Delphi Techniques" OR "Delphi" OR "RAND UCLA appropriateness" OR "RAND appropriateness" OR "UCLA appropriateness") OR AK=("Qualitative Research" OR "Qualitative Research" OR "qualitative studies" OR "qualitative study" OR "qualitative analysis" OR "qualitative" OR "qualitatively" OR "qualitativ*" OR "Focus Groups" OR "Focus Group" OR "Cultural Anthropology" OR "Cultural Anthropology" OR "Material Culture" OR "Ethnography" OR "Interview" OR "Interviews" OR "Interview" OR "Interviewed" OR "Interviewing" OR "Interviewer" OR "Interviewers" OR "Interview*" OR "Empirical Research" OR "Empirical Research" OR "direct observation" OR "direct observations" OR "direct observation*" OR "Delphi Study" OR "Delphi Technique" OR "Delphi Method" OR "Delphi Methods" OR "Delphi Studies" OR "Delphi study" OR "Delphi Technic" OR "Delphi Techniques" OR "Delphi" OR "RAND UCLA appropriateness" OR "RAND appropriateness" OR "UCLA appropriateness") OR AB=("Qualitative Research" OR "Qualitative Research" OR "qualitative studies" OR "qualitative study" OR "qualitative analysis" OR "qualitative" OR "qualitatively" OR "qualitativ*" OR "Focus Groups" OR "Focus Group" OR "Cultural Anthropology" OR "Cultural Anthropology" OR "Material Culture" OR "Ethnography" OR "Interview" OR "Interviews" OR "Interview" OR "Interviewed" OR "Interviewing" OR "Interviewer" OR "Interviewers" OR "Interview*" OR "Empirical Research" OR "Empirical Research" OR "direct observation" OR "direct observations" OR "direct observation*" OR "Delphi Study" OR "Delphi Technique" OR "Delphi Method" OR "Delphi Methods" OR "Delphi Studies" OR "Delphi study" OR "Delphi Technic" OR "Delphi Techniques" OR "Delphi" OR "RAND UCLA appropriateness" OR "RAND appropriateness" OR "UCLA appropriateness")) AND LA=english) OR (TI=("Fracture" OR "Fracture" OR "Fractures" OR "fractur*") AND (TI=("Osteoporosis" OR "Osteoporosis" OR "Osteoporotic" OR "Osteoporo*" OR "Fragility Fracture") OR AK=("Osteoporosis" OR "Osteoporosis" OR "Osteoporotic" OR "Osteoporo*" OR "Fragility Fracture") OR AB=("Osteoporosis" OR "Osteoporosis" OR "Osteoporotic" OR "Osteoporo*" OR "Fragility Fracture")) AND (TI=("Qualitative Research" OR "Qualitative Research" OR "qualitative studies" OR "qualitative study" OR "qualitative analysis" "Focus Groups" OR "Focus Group" OR "Cultural Anthropology" OR "Cultural Anthropology" OR "Material Culture" OR "Ethnography" OR "Interview" OR "Interviews" OR "Interview" OR "Interviewed" OR "Interviewing" OR "Interviewer" OR "Interviewers" OR "Interview*" OR "Empirical Research" OR "Empirical Research" OR "direct observation" OR "direct observations" OR "direct observation*" OR "Delphi Study" OR "Delphi Technique" OR "Delphi Method" OR "Delphi Methods" OR "Delphi Studies" OR "Delphi study" OR "Delphi Technic" OR "Delphi Techniques" OR "Delphi" OR "RAND UCLA appropriateness" OR "RAND appropriateness" OR "UCLA appropriateness") OR AK=("Qualitative Research" OR "Qualitative Research" OR "qualitative studies" OR "qualitative study" OR "qualitative analysis"

"Focus Groups" OR "Focus Group" OR "Cultural Anthropology" OR "Cultural Anthropology" OR "Material Culture" OR "Ethnography" OR "Interview" OR "Interviews" OR "Interview" OR "Interviewed" OR "Interviewing" OR "Interviewer" OR "Interviewers" OR "Interview*" OR "Empirical Research" OR "Empirical Research" OR "direct observation" OR "direct observations" OR "direct observation*" OR "Delphi Study" OR "Delphi Technique" OR "Delphi Method" OR "Delphi Methods" OR "Delphi Studies" OR "Delphi study" OR "Delphi Technic" OR "Delphi Techniques" OR "Delphi" OR "RAND UCLA appropriateness" OR "RAND appropriateness" OR "UCLA appropriateness") OR AB=("Qualitative Research" OR "Qualitative Research" OR "qualitative studies" OR "qualitative study" OR "qualitative analysis" "Focus Groups" OR "Focus Group" OR "Cultural Anthropology" OR "Cultural Anthropology" OR "Material Culture" OR "Ethnography" OR "Interview" OR "Interviews" OR "Interview" OR "Interviewed" OR "Interviewing" OR "Interviewer" OR "Interviewers" OR "Interview*" OR "Empirical Research" OR "Empirical Research" OR "direct observation" OR "direct observations" OR "direct observation*" OR "Delphi Study" OR "Delphi Technique" OR "Delphi Method" OR "Delphi Methods" OR "Delphi Studies" OR "Delphi study" OR "Delphi Technic" OR "Delphi Techniques" OR "Delphi" OR "RAND UCLA appropriateness" OR "RAND appropriateness" OR "UCLA appropriateness") OR TI=("qualitative" OR "qualitatively" OR "qualitativ*")) AND LA=english))

NOT ti=("veterinary" OR "rabbit" OR "rabbits" OR "animal" OR "animals" OR "mouse" OR "mice" OR "rodent" OR "rodents" OR "rat" OR "rats" OR "pig" OR "pigs" OR "porcine" OR "horse" OR "horses" OR "equine" OR "cow" OR "cows" OR "bovine" OR "goat" OR "goats" OR "sheep" OR "ovine" OR "canine" OR "dog" OR "dogs" OR "feline" OR "cat" OR "cats

Search string for Cochrane Library:
((("osteoporotic hip fracture" OR "osteoporotic hip fractures" OR "osteoporosis hip fracture" OR "hip osteoporotic fracture" OR "hip osteoporotic fractures" OR (("osteoporotic" AND "hip" AND "fracture") OR ("osteoporotic" AND "hip" AND "fractures") OR ("osteoporotic" AND "hip" AND "fractured") OR ("osteoporotic" AND "hip" AND "fracturing") OR ("osteoporosis" AND "hip" AND "fracture") OR ("osteoporosis" AND "hip" AND "fractures") OR ("osteoporosis" AND "hip" AND "fractured") OR ("osteoporosis" AND "hip" AND "fracturing")) OR (("Hip Fracture" OR "Hip Fractures" OR "Femoral Neck Fractures" OR "Proximal Femoral Fractures" OR "Intertrochanteric Fractures" OR "Trochanteric Fractures" OR "Subtrochanteric Fractures" OR "Pertrochanteric fractures" OR "Hip Fracture" OR "Femoral Neck Fracture" OR "Proximal Femoral Fracture" OR "Intertrochanteric Fracture" OR "Trochanteric Fracture" OR "Subtrochanteric Fracture" OR "Pertrochanteric fracture" OR (("Hip" OR "Hips") AND ("Fracture" OR "Fractures" OR "fractur*"))) AND ("Osteoporosis" OR "Osteoporosis" OR "Osteoporotic" OR "Osteoporo*" OR "Fragility Fracture")) OR "Fragility Fracture" OR "Fragility fracture" OR "Fragility fractures" OR "Frailty fracture " OR "Frailty fractures" OR (("Fragility" AND "fracture") OR ("Fragility" AND "fractures") OR ("Frailty" AND "fracture") OR ("Frailty" AND "fractures")) OR "Fracture Liaison Service" OR "Fracture Liaison" OR "Osteoporosis Liaison" OR "low trauma fracture" OR "low trauma fractures" OR (("Osteoporosis" AND "Liaison") OR ("Fracture" AND "Liaison") OR ("low" AND "trauma" AND "fracture") OR ("low" AND "trauma" AND "fractures"))) AND ("Qualitative Research" OR "Qualitative Research" OR "qualitative studies" OR "qualitative study" OR "qualitative analysis" OR "qualitative" OR "qualitatively" OR "qualitativ*" OR "Focus Groups" OR "Focus Group" OR "Cultural Anthropology" OR "Cultural Anthropology" OR "Material Culture" OR "Ethnography" OR "Interview" OR "Interviews" OR "Interview" OR "Interviewed" OR "Interviewing" OR "Interviewer" OR "Interviewers" OR "Interview*" OR "Empirical Research" OR "Empirical Research" OR "direct observation" OR "direct observations" OR "direct observation*" OR "Delphi Study" OR "Delphi

Technique" OR "Delphi Method" OR "Delphi Methods" OR "Delphi Studies" OR "Delphi study" OR "Delphi Technic" OR "Delphi Techniques" OR "Delphi" OR "RAND UCLA appropriateness" OR "RAND appropriateness" OR "UCLA appropriateness")):ti,kw OR (("Fracture" OR "Fracture" OR "Fractures" OR "fractur*"):ti AND ("Osteoporosis" OR "Osteoporosis" OR "Osteoporotic" OR "Osteoporo*" OR "Fragility Fracture"):ti,kw AND (("Qualitative Research" OR "Qualitative Research" OR "qualitative studies" OR "qualitative study" OR "qualitative analysis" "Focus Groups" OR "Focus Group" OR "Cultural Anthropology" OR "Cultural Anthropology" OR "Material Culture" OR "Ethnography" OR "Interview" OR "Interviews" OR "Interview" OR "Interviewed" OR "Interviewing" OR "Interviewer" OR "Interviewers" OR "Interview*" OR "Empirical Research" OR "Empirical Research" OR "direct observation" OR "direct observations" OR "direct observation*" OR "Delphi Study" OR "Delphi Technique" OR "Delphi Method" OR "Delphi Methods" OR "Delphi Studies" OR "Delphi study" OR "Delphi Technic" OR "Delphi Techniques" OR "Delphi" OR "RAND UCLA appropriateness" OR "RAND appropriateness" OR "UCLA appropriateness"):ti,kw OR ("qualitative" OR "qualitatively" OR "qualitativ*"):ti))) OR ((("osteoporotic hip fracture" OR "osteoporotic hip fractures" OR "osteoporosis hip fracture" OR "hip osteoporotic fracture" OR "hip osteoporotic fractures" OR (("osteoporotic" NEAR/0 "hip" NEAR/0 "fracture") OR ("osteoporotic" NEAR/0 "hip" NEAR/0 "fractures") OR ("osteoporotic" NEAR/0 "hip" NEAR/0 "fractured") OR ("osteoporotic" NEAR/0 "hip" NEAR/0 "fracturing") OR ("osteoporosis" NEAR/0 "hip" NEAR/0 "fracture") OR ("osteoporosis" NEAR/0 "hip" NEAR/0 "fractures") OR ("osteoporosis" NEAR/0 "hip" NEAR/0 "fractured") OR ("osteoporosis" NEAR/0 "hip" NEAR/0 "fracturing")) OR (("Hip Fracture" OR "Hip Fractures" OR "Femoral Neck Fractures" OR "Proximal Femoral Fractures" OR "Intertrochanteric Fractures" OR "Trochanteric Fractures" OR "Subtrochanteric Fractures" OR "Pertrochanteric fractures" OR "Hip Fracture" OR "Femoral Neck Fracture" OR "Proximal Femoral Fracture" OR "Intertrochanteric Fracture" OR "Trochanteric Fracture" OR "Subtrochanteric Fracture" OR "Pertrochanteric fracture" OR (("Hip" OR "Hips") AND ("Fracture" OR "Fractures" OR "fractur*"))) AND ("Osteoporosis" OR "Osteoporosis" OR "Osteoporotic" OR "Osteoporo*" OR "Fragility Fracture")) OR "Fragility Fracture" OR "Fragility fracture" OR "Fragility fractures" OR "Frailty fracture " OR "Frailty fractures" OR (("Fragility" NEAR/0 "fracture") OR ("Fragility" NEAR/0 "fractures") OR ("Frailty" NEAR/0 "fracture") OR ("Frailty" NEAR/0 "fractures")) OR "Fracture Liaison Service" OR "Fracture Liaison" OR "Osteoporosis Liaison" OR "low trauma fracture" OR "low trauma fractures" OR (("Osteoporosis" NEAR/0 "Liaison") OR ("Fracture" NEAR/0 "Liaison") OR ("low" NEAR/0 "trauma" NEAR/0 "fracture") OR ("low" NEAR/0 "trauma" NEAR/0 "fractures"))) AND ("Qualitative Research" OR "Qualitative Research" OR "qualitative studies" OR "qualitative study" OR "qualitative analysis" OR "qualitative" OR "qualitatively" OR "qualitativ*" OR "Focus Groups" OR "Focus Group" OR "Cultural Anthropology" OR "Cultural Anthropology" OR "Material Culture" OR "Ethnography" OR "Interview" OR "Interviews" OR "Interview" OR "Interviewed" OR "Interviewing" OR "Interviewer" OR "Interviewers" OR "Interview*" OR "Empirical Research" OR "Empirical Research" OR "direct observation" OR "direct observations" OR "direct observation*" OR "Delphi Study" OR "Delphi Technique" OR "Delphi Method" OR "Delphi Methods" OR "Delphi Studies" OR "Delphi study" OR "Delphi Technic" OR "Delphi Techniques" OR "Delphi" OR "RAND UCLA appropriateness" OR "RAND appropriateness" OR "UCLA appropriateness")):ti,kw OR (("Fracture" OR "Fracture" OR "Fractures" OR "fractur*"):ti AND ("Osteoporosis" OR "Osteoporosis" OR "Osteoporotic" OR "Osteoporo*" OR "Fragility Fracture"):ti,kw AND (("Qualitative Research" OR "Qualitative Research" OR "qualitative studies" OR "qualitative study" OR "qualitative analysis" "Focus Groups" OR "Focus Group" OR "Cultural Anthropology" OR "Cultural Anthropology" OR "Material Culture" OR "Ethnography" OR "Interview" OR "Interviews" OR "Interview" OR "Interviewed" OR "Interviewing" OR "Interviewer" OR "Interviewers" OR "Interview*" OR "Empirical Research" OR "Empirical Research" OR "direct observation" OR "direct observations" OR "direct observation*" OR "Delphi Study" OR "Delphi Technique" OR "Delphi Method" OR "Delphi Methods" OR "Delphi Studies" OR "Delphi study" OR "Delphi Technic" OR "Delphi Techniques" OR "Delphi" OR "RAND UCLA appropriateness" OR

"RAND appropriateness" OR "UCLA appropriateness"):ti,kw OR ("qualitative" OR "qualitatively" OR "qualitativ*"):ti)))

(conference abstract OR meeting abstract OR conference proceeding OR conference proceedings):pt

Search string for PsycINFO:
Limit: language = english

(((TI("osteoporotic hip fracture" OR "osteoporotic hip fractures" OR "osteoporosis hip fracture" OR "hip osteoporotic fracture" OR "hip osteoporotic fractures" OR ("osteoporotic" AND "hip" AND "fracture") OR ("osteoporotic" AND "hip" AND "fractures") OR ("osteoporotic" AND "hip" AND "fractured") OR ("osteoporotic" AND "hip" AND "fracturing") OR ("osteoporosis" AND "hip" AND "fracture") OR ("osteoporosis" AND "hip" AND "fractures") OR ("osteoporosis" AND "hip" AND "fractured") OR ("osteoporosis" AND "hip" AND "fracturing") OR (("Hip Fracture" OR "Hip Fractures" OR "Femoral Neck Fractures" OR "Proximal Femoral Fractures" OR "Intertrochanteric Fractures" OR "Trochanteric Fractures" OR "Subtrochanteric Fractures" OR "Pertrochanteric fractures" OR "Hip Fracture" OR "Femoral Neck Fracture" OR "Proximal Femoral Fracture" OR "Intertrochanteric Fracture" OR "Trochanteric Fracture" OR "Subtrochanteric Fracture" OR "Pertrochanteric fracture" OR (("Hip" OR "Hips") AND ("Fracture" OR "Fractures" OR "fractur*"))) AND ("Osteoporosis" OR "Osteoporosis" OR "Osteoporotic" OR "Osteoporo*" OR "Fragility Fracture")) OR "Fragility Fracture" OR "Fragility fracture" OR "Fragility fractures" OR "Frailty fracture " OR "Frailty fractures" OR ("Fragility" AND "fracture") OR ("Fragility" AND "fractures") OR ("Frailty" AND "fracture") OR ("Frailty" AND "fractures") OR "Fracture Liaison Service" OR "Fracture Liaison" OR "Osteoporosis Liaison" OR "low trauma fracture" OR "low trauma fractures" OR ("Osteoporosis" AND "Liaison") OR ("Fracture" AND "Liaison") OR ("low" AND "trauma" AND "fracture") OR ("low" AND "trauma" AND "fractures")) OR SU("osteoporotic hip fracture" OR "osteoporotic hip fractures" OR "osteoporosis hip fracture" OR "hip osteoporotic fracture" OR "hip osteoporotic fractures" OR ("osteoporotic" AND "hip" AND "fracture") OR ("osteoporotic" AND "hip" AND "fractures") OR ("osteoporotic" AND "hip" AND "fractured") OR ("osteoporotic" AND "hip" AND "fracturing") OR ("osteoporosis" AND "hip" AND "fracture") OR ("osteoporosis" AND "hip" AND "fractures") OR ("osteoporosis" AND "hip" AND "fractured") OR ("osteoporosis" AND "hip" AND "fracturing") OR (("Hip Fracture" OR "Hip Fractures" OR "Femoral Neck Fractures" OR "Proximal Femoral Fractures" OR "Intertrochanteric Fractures" OR "Trochanteric Fractures" OR "Subtrochanteric Fractures" OR "Pertrochanteric fractures" OR "Hip Fracture" OR "Femoral Neck Fracture" OR "Proximal Femoral Fracture" OR "Intertrochanteric Fracture" OR "Trochanteric Fracture" OR "Subtrochanteric Fracture" OR "Pertrochanteric fracture" OR (("Hip" OR "Hips") AND ("Fracture" OR "Fractures" OR "fractur*"))) AND ("Osteoporosis" OR "Osteoporosis" OR "Osteoporotic" OR "Osteoporo*" OR "Fragility Fracture")) OR "Fragility Fracture" OR "Fragility fracture" OR "Fragility fractures" OR "Frailty fracture " OR "Frailty fractures" OR ("Fragility" AND "fracture") OR ("Fragility" AND "fractures") OR ("Frailty" AND "fracture") OR ("Frailty" AND "fractures") OR "Fracture Liaison Service" OR "Fracture Liaison" OR "Osteoporosis Liaison" OR "low trauma fracture" OR "low trauma fractures" OR ("Osteoporosis" AND "Liaison") OR ("Fracture" AND "Liaison") OR ("low" AND "trauma" AND "fracture") OR ("low" AND "trauma" AND "fractures")) OR AB("osteoporotic hip fracture" OR "osteoporotic hip fractures" OR "osteoporosis hip fracture" OR "hip osteoporotic fracture" OR "hip osteoporotic fractures" OR ("osteoporotic" AND "hip" AND "fracture") OR ("osteoporotic" AND "hip" AND "fractures") OR ("osteoporotic" AND "hip" AND "fractured") OR ("osteoporotic" AND "hip" AND "fracturing") OR ("osteoporosis" AND "hip" AND "fracture") OR ("osteoporosis" AND "hip" AND "fractures") OR ("osteoporosis" AND "hip" AND "fractured") OR ("osteoporosis" AND "hip" AND "fracturing") OR (("Hip Fracture" OR "Hip Fractures" OR "Femoral Neck Fractures" OR "Proximal Femoral Fractures" OR "Intertrochanteric Fractures" OR "Trochanteric Fractures" OR "Subtrochanteric Fractures" OR "Pertrochanteric fractures" OR "Hip Fracture" OR "Femoral Neck Fracture" OR

"Proximal Femoral Fracture" OR "Intertrochanteric Fracture" OR "Trochanteric Fracture" OR "Subtrochanteric Fracture" OR "Pertrochanteric fracture" OR (("Hip" OR "Hips") AND ("Fracture" OR "Fractures" OR "fractur*"))) AND ("Osteoporosis" OR "Osteoporosis" OR "Osteoporotic" OR "Osteoporo*" OR "Fragility Fracture")) OR "Fragility Fracture" OR "Fragility fracture" OR "Fragility fractures" OR "Frailty fracture " OR "Frailty fractures" OR ("Fragility" AND "fracture") OR ("Fragility" AND "fractures") OR ("Frailty" AND "fracture") OR ("Frailty" AND "fractures") OR "Fracture Liaison Service" OR "Fracture Liaison" OR "Osteoporosis Liaison" OR "low trauma fracture" OR "low trauma fractures" OR ("Osteoporosis" AND "Liaison") OR ("Fracture" AND "Liaison") OR ("low" AND "trauma" AND "fracture") OR ("low" AND "trauma" AND "fractures"))) AND (TI("Qualitative Research" OR "Qualitative Research" OR "qualitative studies" OR "qualitative study" OR "qualitative analysis" OR "qualitative" OR "qualitatively" OR "qualitativ*" OR "Focus Groups" OR "Focus Group" OR "Cultural Anthropology" OR "Cultural Anthropology" OR "Material Culture" OR "Ethnography" OR "Interview" OR "Interviews" OR "Interview" OR "Interviewed" OR "Interviewing" OR "Interviewer" OR "Interviewers" OR "Interview*" OR "Empirical Research" OR "Empirical Research" OR "direct observation" OR "direct observations" OR "direct observation*" OR "Delphi Study" OR "Delphi Technique" OR "Delphi Method" OR "Delphi Methods" OR "Delphi Studies" OR "Delphi study" OR "Delphi Technic" OR "Delphi Techniques" OR "Delphi" OR "RAND UCLA appropriateness" OR "RAND appropriateness" OR "UCLA appropriateness") OR SU("Qualitative Research" OR "Qualitative Research" OR "qualitative studies" OR "qualitative study" OR "qualitative analysis" OR "qualitative" OR "qualitatively" OR "qualitativ*" OR "Focus Groups" OR "Focus Group" OR "Cultural Anthropology" OR "Cultural Anthropology" OR "Material Culture" OR "Ethnography" OR "Interview" OR "Interviews" OR "Interview" OR "Interviewed" OR "Interviewing" OR "Interviewer" OR "Interviewers" OR "Interview*" OR "Empirical Research" OR "Empirical Research" OR "direct observation" OR "direct observations" OR "direct observation*" OR "Delphi Study" OR "Delphi Technique" OR "Delphi Method" OR "Delphi Methods" OR "Delphi Studies" OR "Delphi study" OR "Delphi Technic" OR "Delphi Techniques" OR "Delphi" OR "RAND UCLA appropriateness" OR "RAND appropriateness" OR "UCLA appropriateness") OR AB("Qualitative Research" OR "Qualitative Research" OR "qualitative studies" OR "qualitative study" OR "qualitative analysis" OR "qualitative" OR "qualitatively" OR "qualitativ*" OR "Focus Groups" OR "Focus Group" OR "Cultural Anthropology" OR "Cultural Anthropology" OR "Material Culture" OR "Ethnography" OR "Interview" OR "Interviews" OR "Interview" OR "Interviewed" OR "Interviewing" OR "Interviewer" OR "Interviewers" OR "Interview*" OR "Empirical Research" OR "Empirical Research" OR "direct observation" OR "direct observations" OR "direct observation*" OR "Delphi Study" OR "Delphi Technique" OR "Delphi Method" OR "Delphi Methods" OR "Delphi Studies" OR "Delphi study" OR "Delphi Technic" OR "Delphi Techniques" OR "Delphi" OR "RAND UCLA appropriateness" OR "RAND appropriateness" OR "UCLA appropriateness") OR MR("Qualitative Research" OR "Qualitative Research" OR "qualitative studies" OR "qualitative study" OR "qualitative analysis" OR "qualitative" OR "qualitatively" OR "qualitativ*" OR "Focus Groups" OR "Focus Group" OR "Cultural Anthropology" OR "Cultural Anthropology" OR "Material Culture" OR "Ethnography" OR "Interview" OR "Interviews" OR "Interview" OR "Interviewed" OR "Interviewing" OR "Interviewer" OR "Interviewers" OR "Interview*" OR "Empirical Research" OR "Empirical Research" OR "direct observation" OR "direct observations" OR "direct observation*" OR "Delphi Study" OR "Delphi Technique" OR "Delphi Method" OR "Delphi Methods" OR "Delphi Studies" OR "Delphi study" OR "Delphi Technic" OR "Delphi Techniques" OR "Delphi" OR "RAND UCLA appropriateness" OR "RAND appropriateness" OR "UCLA appropriateness"))) OR (TI("Fracture" OR "Fracture" OR "Fractures" OR "fractur*") AND (TI("Osteoporosis" OR "Osteoporosis" OR "Osteoporotic" OR "Osteoporo*" OR "Fragility Fracture") OR SU("Osteoporosis" OR "Osteoporosis" OR "Osteoporotic" OR "Osteoporo*" OR "Fragility Fracture") OR AB("Osteoporosis" OR "Osteoporosis" OR "Osteoporotic" OR "Osteoporo*" OR "Fragility Fracture")) AND (TI("Qualitative Research" OR "Qualitative Research" OR "qualitative studies" OR "qualitative study" OR "qualitative analysis" "Focus Groups" OR "Focus Group" OR "Cultural Anthropology" OR "Cultural Anthropology" OR "Material Culture" OR "Ethnography" OR "Interview" OR "Interviews" OR "Interview" OR "Interviewed" OR "Interviewing" OR "Interviewer" OR "Interviewers" OR "Interview*" OR "Empirical Research" OR "Empirical Research" OR "direct observation" OR "direct observations" OR "direct observation*" OR "Delphi Study" OR "Delphi

Technique" OR "Delphi Method" OR "Delphi Methods" OR "Delphi Studies" OR "Delphi study" OR "Delphi Technic" OR "Delphi Techniques" OR "Delphi" OR "RAND UCLA appropriateness" OR "RAND appropriateness" OR "UCLA appropriateness") OR SU("Qualitative Research" OR "Qualitative Research" OR "qualitative studies" OR "qualitative study" OR "qualitative analysis" "Focus Groups" OR "Focus Group" OR "Cultural Anthropology" OR "Cultural Anthropology" OR "Material Culture" OR "Ethnography" OR "Interview" OR "Interviews" OR "Interview" OR "Interviewed" OR "Interviewing" OR "Interviewer" OR "Interviewers" OR "Interview*" OR "Empirical Research" OR "Empirical Research" OR "direct observation" OR "direct observations" OR "direct observation*" OR "Delphi Study" OR "Delphi Technique" OR "Delphi Method" OR "Delphi Methods" OR "Delphi Studies" OR "Delphi study" OR "Delphi Technic" OR "Delphi Techniques" OR "Delphi" OR "RAND UCLA appropriateness" OR "RAND appropriateness" OR "UCLA appropriateness") OR AB("Qualitative Research" OR "Qualitative Research" OR "qualitative studies" OR "qualitative study" OR "qualitative analysis" "Focus Groups" OR "Focus Group" OR "Cultural Anthropology" OR "Cultural Anthropology" OR "Material Culture" OR "Ethnography" OR "Interview" OR "Interviews" OR "Interview" OR "Interviewed" OR "Interviewing" OR "Interviewer" OR "Interviewers" OR "Interview*" OR "Empirical Research" OR "Empirical Research" OR "direct observation" OR "direct observations" OR "direct observation*" OR "Delphi Study" OR "Delphi Technique" OR "Delphi Method" OR "Delphi Methods" OR "Delphi Studies" OR "Delphi study" OR "Delphi Technic" OR "Delphi Techniques" OR "Delphi" OR "RAND UCLA appropriateness" OR "RAND appropriateness" OR "UCLA appropriateness") OR MR("Qualitative Research" OR "Qualitative Research" OR "qualitative studies" OR "qualitative study" OR "qualitative analysis" OR "qualitative" OR "qualitatively" OR "qualitativ*" OR "Focus Groups" OR "Focus Group" OR "Cultural Anthropology" OR "Cultural Anthropology" OR "Material Culture" OR "Ethnography" OR "Interview" OR "Interviews" OR "Interview" OR "Interviewed" OR "Interviewing" OR "Interviewer" OR "Interviewers" OR "Interview*" OR "Empirical Research" OR "Empirical Research" OR "direct observation" OR "direct observations" OR "direct observation*" OR "Delphi Study" OR "Delphi Technique" OR "Delphi Method" OR "Delphi Methods" OR "Delphi Studies" OR "Delphi study" OR "Delphi Technic" OR "Delphi Techniques" OR "Delphi" OR "RAND UCLA appropriateness" OR "RAND appropriateness" OR "UCLA appropriateness")OR TI("qualitative" OR "qualitatively" OR "qualitativ*"))))

Search string for Emcare:
((("osteoporotic hip fracture".ti,ab OR "osteoporotic hip fractures".ti,ab OR "osteoporosis hip fracture".ti,ab OR "hip osteoporotic fracture".ti,ab OR "hip osteoporotic fractures".ti,ab OR (("osteoporotic" AND "hip" AND "fracture") OR ("osteoporotic" AND "hip" AND "fractures") OR ("osteoporotic" AND "hip" AND "fractured") OR ("osteoporotic" AND "hip" AND "fracturing") OR ("osteoporosis" AND "hip" AND "fracture") OR ("osteoporosis" AND "hip" AND "fractures") OR ("osteoporosis" AND "hip" AND "fractured") OR ("osteoporosis" AND "hip" AND "fracturing")).ti,ab OR (exp *"Hip Fracture"/ AND exp *"Fragility Fracture"/) OR (exp *"Hip Fractures"/ AND exp *"Osteoporosis"/) OR ((exp *"Hip Fracture"/ OR "Hip Fractures".ti,ab OR "Femoral Neck Fractures".ti,ab OR "Proximal Femoral Fractures".ti,ab OR "Intertrochanteric Fractures".ti,ab OR "Trochanteric Fractures".ti,ab OR "Subtrochanteric Fractures".ti,ab OR "Pertrochanteric fractures".ti,ab OR "Hip Fracture".ti,ab OR "Femoral Neck Fracture".ti,ab OR "Proximal Femoral Fracture".ti,ab OR "Intertrochanteric Fracture".ti,ab OR "Trochanteric Fracture".ti,ab OR "Subtrochanteric Fracture".ti,ab OR "Pertrochanteric fracture".ti,ab OR (("Hip".ti,ab OR "Hips".ti,ab) AND ("Fracture".ti,ab OR "Fractures".ti,ab OR "fractur*".ti,ab))) AND (exp *"Osteoporosis"/ OR "Osteoporosis".ti,ab OR "Osteoporotic".ti,ab OR "Osteoporo*".ti,ab OR exp *"Fragility Fracture"/)) OR exp *"Fragility Fracture"/ OR "Fragility fracture".ti,ab OR "Fragility fractures".ti,ab OR "Frailty fracture ".ti,ab OR "Frailty fractures".ti,ab OR (("Fragility" AND "fracture") OR ("Fragility" AND "fractures") OR ("Frailty" AND "fracture") OR ("Frailty" AND "fractures")).ti,ab OR "Fracture Liaison Service".ti,ab OR "Fracture Liaison".ti,ab OR "Osteoporosis Liaison".ti,ab OR "low trauma fracture".ti,ab OR "low trauma fractures".ti,ab OR (("Osteoporosis"

AND "Liaison") OR ("Fracture" AND "Liaison") OR ("low" AND "trauma" AND "fracture") OR ("low" AND "trauma" AND "fractures")).ti,ab) AND (exp *"Qualitative Research"/ OR "Qualitative Research".ti,ab OR "qualitative studies".ti,ab OR "qualitative study".ti,ab OR "qualitative analysis".ti,ab OR "qualitative".ti,ab OR "qualitatively".ti,ab OR "qualitativ*".ti,ab OR "Focus Groups".ti,ab OR "Focus Group".ti,ab OR exp *"Cultural Anthropology"/ OR "Cultural Anthropology".ti,ab OR "Material Culture".ti,ab OR "Ethnography".ti,ab OR exp *"Interview"/ OR "Interviews".ti,ab OR "Interview".ti,ab OR "Interviewed".ti,ab OR "Interviewing".ti,ab OR "Interviewer".ti,ab OR "Interviewers".ti,ab OR "Interview*".ti,ab OR exp *"Empirical Research"/ OR "Empirical Research".ti,ab OR "direct observation".ti,ab OR "direct observations".ti,ab OR "direct observation*".ti,ab OR exp *"Delphi Study"/ OR "Delphi Technique".ti,ab OR "Delphi Method".ti,ab OR "Delphi Methods".ti,ab OR "Delphi Studies".ti,ab OR "Delphi study".ti,ab OR "Delphi Technic".ti,ab OR "Delphi Techniques".ti,ab OR "Delphi".ti,ab OR "RAND UCLA appropriateness".ti,ab OR "RAND appropriateness".ti,ab OR "UCLA appropriateness".ti,ab) AND english.la) OR ((exp *"Fracture"/ OR "Fracture".ti OR "Fractures".ti OR "fractur*".ti) AND (exp *"Osteoporosis"/ OR "Osteoporosis".ti,ab OR "Osteoporotic".ti,ab OR "Osteoporo*".ti,ab OR exp *"Fragility Fracture"/) AND (exp *"Qualitative Research"/ OR "Qualitative Research".ti,ab OR "qualitative studies".ti,ab OR "qualitative study".ti,ab OR "qualitative analysis".ti,ab OR "qualitative".ti OR "qualitatively".ti OR "qualitativ*".ti OR "Focus Groups".ti,ab OR "Focus Group".ti,ab OR exp *"Cultural Anthropology"/ OR "Cultural Anthropology".ti,ab OR "Material Culture".ti,ab OR "Ethnography".ti,ab OR exp *"Interview"/ OR "Interviews".ti,ab OR "Interview".ti,ab OR "Interviewed".ti,ab OR "Interviewing".ti,ab OR "Interviewer".ti,ab OR "Interviewers".ti,ab OR "Interview*".ti,ab OR exp *"Empirical Research"/ OR "Empirical Research".ti,ab OR "direct observation".ti,ab OR "direct observations".ti,ab OR "direct observation*".ti,ab OR exp *"Delphi Study"/ OR "Delphi Technique".ti,ab OR "Delphi Method".ti,ab OR "Delphi Methods".ti,ab OR "Delphi Studies".ti,ab OR "Delphi study".ti,ab OR "Delphi Technic".ti,ab OR "Delphi Techniques".ti,ab OR "Delphi".ti,ab OR "RAND UCLA appropriateness".ti,ab OR "RAND appropriateness".ti,ab OR "UCLA appropriateness".ti,ab) AND english.la)**)**

Search string for Academic Search Premier

Limit language = english

((TI("osteoporotic hip fracture" OR "osteoporotic hip fractures" OR "osteoporosis hip fracture" OR "hip osteoporotic fracture" OR "hip osteoporotic fractures" OR ("osteoporotic" AND "hip" AND "fracture") OR ("osteoporotic" AND "hip" AND "fractures") OR ("osteoporotic" AND "hip" AND "fractured") OR ("osteoporotic" AND "hip" AND "fracturing") OR ("osteoporosis" AND "hip" AND "fracture") OR ("osteoporosis" AND "hip" AND "fractures") OR ("osteoporosis" AND "hip" AND "fractured") OR ("osteoporosis" AND "hip" AND "fracturing") OR (("Hip Fracture" OR "Hip Fractures" OR "Femoral Neck Fractures" OR "Proximal Femoral Fractures" OR "Intertrochanteric Fractures" OR "Trochanteric Fractures" OR "Subtrochanteric Fractures" OR "Pertrochanteric fractures" OR "Hip Fracture" OR "Femoral Neck Fracture" OR "Proximal Femoral Fracture" OR "Intertrochanteric Fracture" OR "Trochanteric Fracture" OR "Subtrochanteric Fracture" OR "Pertrochanteric fracture" OR (("Hip" OR "Hips") AND ("Fracture" OR "Fractures" OR "fractur*"))) AND ("Osteoporosis" OR "Osteoporosis" OR "Osteoporotic" OR "Osteoporo*" OR "Fragility Fracture")) OR "Fragility Fracture" OR "Fragility fracture" OR "Fragility fractures" OR "Frailty fracture " OR "Frailty fractures" OR ("Fragility" AND "fracture") OR ("Fragility" AND "fractures") OR ("Frailty" AND "fracture") OR ("Frailty" AND "fractures") OR "Fracture Liaison Service" OR "Fracture Liaison" OR "Osteoporosis Liaison" OR "low trauma fracture" OR "low trauma fractures" OR ("Osteoporosis" AND "Liaison") OR ("Fracture" AND "Liaison") OR ("low" AND "trauma" AND "fracture") OR ("low" AND "trauma" AND "fractures")) OR KW("osteoporotic hip fracture" OR

"osteoporotic hip fractures" OR "osteoporosis hip fracture" OR "hip osteoporotic fracture" OR "hip osteoporotic fractures" OR ("osteoporotic" AND "hip" AND "fracture") OR ("osteoporotic" AND "hip" AND "fractures") OR ("osteoporotic" AND "hip" AND "fractured") OR ("osteoporotic" AND "hip" AND "fracturing") OR ("osteoporosis" AND "hip" AND "fracture") OR ("osteoporosis" AND "hip" AND "fractures") OR ("osteoporosis" AND "hip" AND "fractured") OR ("osteoporosis" AND "hip" AND "fracturing") OR (("Hip Fracture" OR "Hip Fractures" OR "Femoral Neck Fractures" OR "Proximal Femoral Fractures" OR "Intertrochanteric Fractures" OR "Trochanteric Fractures" OR "Subtrochanteric Fractures" OR "Pertrochanteric fractures" OR "Hip Fracture" OR "Femoral Neck Fracture" OR "Proximal Femoral Fracture" OR "Intertrochanteric Fracture" OR "Trochanteric Fracture" OR "Subtrochanteric Fracture" OR "Pertrochanteric fracture" OR (("Hip" OR "Hips") AND ("Fracture" OR "Fractures" OR "fractur*"))) AND ("Osteoporosis" OR "Osteoporosis" OR "Osteoporotic" OR "Osteoporo*" OR "Fragility Fracture")) OR "Fragility Fracture" OR "Fragility fracture" OR "Fragility fractures" OR "Frailty fracture " OR "Frailty fractures" OR ("Fragility" AND "fracture") OR ("Fragility" AND "fractures") OR ("Frailty" AND "fracture") OR ("Frailty" AND "fractures") OR "Fracture Liaison Service" OR "Fracture Liaison" OR "Osteoporosis Liaison" OR "low trauma fracture" OR "low trauma fractures" OR ("Osteoporosis" AND "Liaison") OR ("Fracture" AND "Liaison") OR ("low" AND "trauma" AND "fracture") OR ("low" AND "trauma" AND "fractures")) OR SU("osteoporotic hip fracture" OR "osteoporotic hip fractures" OR "osteoporosis hip fracture" OR "hip osteoporotic fracture" OR "hip osteoporotic fractures" OR ("osteoporotic" AND "hip" AND "fracture") OR ("osteoporotic" AND "hip" AND "fractures") OR ("osteoporotic" AND "hip" AND "fractured") OR ("osteoporotic" AND "hip" AND "fracturing") OR ("osteoporosis" AND "hip" AND "fracture") OR ("osteoporosis" AND "hip" AND "fractures") OR ("osteoporosis" AND "hip" AND "fractured") OR ("osteoporosis" AND "hip" AND "fracturing") OR (("Hip Fracture" OR "Hip Fractures" OR "Femoral Neck Fractures" OR "Proximal Femoral Fractures" OR "Intertrochanteric Fractures" OR "Trochanteric Fractures" OR "Subtrochanteric Fractures" OR "Pertrochanteric fractures" OR "Hip Fracture" OR "Femoral Neck Fracture" OR "Proximal Femoral Fracture" OR "Intertrochanteric Fracture" OR "Trochanteric Fracture" OR "Subtrochanteric Fracture" OR "Pertrochanteric fracture" OR (("Hip" OR "Hips") AND ("Fracture" OR "Fractures" OR "fractur*"))) AND ("Osteoporosis" OR "Osteoporosis" OR "Osteoporotic" OR "Osteoporo*" OR "Fragility Fracture")) OR "Fragility Fracture" OR "Fragility fracture" OR "Fragility fractures" OR "Frailty fracture " OR "Frailty fractures" OR ("Fragility" AND "fracture") OR ("Fragility" AND "fractures") OR ("Frailty" AND "fracture") OR ("Frailty" AND "fractures") OR "Fracture Liaison Service" OR "Fracture Liaison" OR "Osteoporosis Liaison" OR "low trauma fracture" OR "low trauma fractures" OR ("Osteoporosis" AND "Liaison") OR ("Fracture" AND "Liaison") OR ("low" AND "trauma" AND "fracture") OR ("low" AND "trauma" AND "fractures"))) AND (TI("Qualitative Research" OR "Qualitative Research" OR "qualitative studies" OR "qualitative study" OR "qualitative analysis" OR "qualitative" OR "qualitatively" OR "qualitativ*" OR "Focus Groups" OR "Focus Group" OR "Cultural Anthropology" OR "Cultural Anthropology" OR "Material Culture" OR "Ethnography" OR "Interview" OR "Interviews" OR "Interview" OR "Interviewed" OR "Interviewing" OR "Interviewer" OR "Interviewers" OR "Interview*" OR "Empirical Research" OR "Empirical Research" OR "direct observation" OR "direct observations" OR "direct observation*" OR "Delphi Study" OR "Delphi Technique" OR "Delphi Method" OR "Delphi Methods" OR "Delphi Studies" OR "Delphi study" OR "Delphi Technic" OR "Delphi Techniques" OR "Delphi" OR "RAND UCLA appropriateness" OR "RAND appropriateness" OR "UCLA appropriateness") OR SU("Qualitative Research" OR "Qualitative Research" OR "qualitative studies" OR "qualitative study" OR "qualitative analysis" OR "qualitative" OR "qualitatively" OR "qualitativ*" OR "Focus Groups" OR "Focus Group" OR "Cultural Anthropology" OR "Cultural Anthropology" OR "Material Culture" OR "Ethnography" OR "Interview" OR "Interviews" OR "Interview" OR "Interviewed" OR "Interviewing" OR "Interviewer" OR "Interviewers" OR "Interview*" OR "Empirical Research" OR "Empirical Research" OR "direct observation" OR "direct observations" OR "direct observation*" OR "Delphi Study" OR "Delphi Technique" OR "Delphi Method" OR "Delphi Methods" OR "Delphi

Studies" OR "Delphi study" OR "Delphi Technic" OR "Delphi Techniques" OR "Delphi" OR "RAND UCLA appropriateness" OR "RAND appropriateness" OR "UCLA appropriateness") OR AB("Qualitative Research" OR "Qualitative Research" OR "qualitative studies" OR "qualitative study" OR "qualitative analysis" OR "qualitative" OR "qualitatively" OR "qualitativ*" OR "Focus Groups" OR "Focus Group" OR "Cultural Anthropology" OR "Cultural Anthropology" OR "Material Culture" OR "Ethnography" OR "Interview" OR "Interviews" OR "Interview" OR "Interviewed" OR "Interviewing" OR "Interviewer" OR "Interviewers" OR "Interview*" OR "Empirical Research" OR "Empirical Research" OR "direct observation" OR "direct observations" OR "direct observation*" OR "Delphi Study" OR "Delphi Technique" OR "Delphi Method" OR "Delphi Methods" OR "Delphi Studies" OR "Delphi study" OR "Delphi Technic" OR "Delphi Techniques" OR "Delphi" OR "RAND UCLA appropriateness" OR "RAND appropriateness" OR "UCLA appropriateness") OR KW("Qualitative Research" OR "Qualitative Research" OR "qualitative studies" OR "qualitative study" OR "qualitative analysis" OR "qualitative" OR "qualitatively" OR "qualitativ*" OR "Focus Groups" OR "Focus Group" OR "Cultural Anthropology" OR "Cultural Anthropology" OR "Material Culture" OR "Ethnography" OR "Interview" OR "Interviews" OR "Interview" OR "Interviewed" OR "Interviewing" OR "Interviewer" OR "Interviewers" OR "Interview*" OR "Empirical Research" OR "Empirical Research" OR "direct observation" OR "direct observations" OR "direct observation*" OR "Delphi Study" OR "Delphi Technique" OR "Delphi Method" OR "Delphi Methods" OR "Delphi Studies" OR "Delphi study" OR "Delphi Technic" OR "Delphi Techniques" OR "Delphi" OR "RAND UCLA appropriateness" OR "RAND appropriateness" OR "UCLA appropriateness")))

Appendix 5: References

1. Bennett MJ, Center JR, Perry L. Exploring barriers and opportunities to improve osteoporosis care across the acute-to-primary care interface: a qualitative study. *Osteoporos Int* 2023;34(7):1249-62. doi: 10.1007/s00198-023-06748-0
2. Bishop S, Narayanasamy MJ, Paskins Z, et al. Clinicians' views of prescribing oral and intravenous bisphosphonates for osteoporosis: a qualitative study. *BMC Musculoskelet Disord* 2023;24(1):770. doi: 10.1186/s12891-023-06865-1
3. Bullock L, Manning F, Hawarden A, et al. Exploring practice and perspectives on shared decision-making about osteoporosis medicines in Fracture Liaison Services: the iFraP development qualitative study. *Arch Osteoporos* 2024;19(1):50. doi: 10.1007/s11657-024-01410-6
4. Cheah MH, Lai PSM, Ong T. Views of healthcare professionals regarding barriers and facilitators for a Fracture Liaison Service in Malaysia. *PLoS One* 2024;19(7):e0307919. doi: 10.1371/journal.pone.0307919
5. Feldstein AC, Schneider J, Smith DH, et al. Harnessing stakeholder perspectives to improve the care of osteoporosis after a fracture. *Osteoporos Int* 2008;19(11):1527-40. doi: 10.1007/s00198-008-0605-3
6. Lerner ET, MacLean KG, Colón-Emeric C, et al. Enabling osteoporosis treatment in post-acute care: An algorithm for providers. *Geriatr Nurs* 2025;61:228-30. doi: 10.1016/j.gerinurse.2024.10.068
7. Otmar R, Reventlow SD, Nicholson GC, et al. General medical practitioners' knowledge and beliefs about osteoporosis and its investigation and management. *Arch Osteoporos* 2012;7:107-14. doi: 10.1007/s11657-012-0088-x
8. Salminen H, Piispanen P, Toth-Pal E. Primary care physicians' views on osteoporosis management: a qualitative study. *Arch Osteoporos* 2019;14(1):48. doi: 10.1007/s11657-019-0599-9
9. Verdonck C, Van Daele E, Willems R, et al. Underlying motivations hampering Flemish primary care physicians from overcoming the barriers in osteoporosis care: an EMR-facilitated clinical reasoning study. *BMC Health Serv Res* 2023;23(1):1428. doi: 10.1186/s12913-023-10441-7
10. Wozniak LA, Beaupre LA, Juby A, et al. Successful implementation of a Fracture Liaison Service through effective change management: a qualitative study. *Arch Osteoporos* 2020;15(1):44. doi: 10.1007/s11657-020-0692-0
11. Drew S, Judge A, Cooper C, et al. Secondary prevention of fractures after hip fracture: a qualitative study of effective service delivery. *Osteoporos Int* 2016;27(5):1719-27. doi: 10.1007/s00198-015-3452-z
12. Guillemot JR, Abraham JW, Tinker A. Understanding trends in osteoporosis drug prescribing: implications for reducing futile biomedical research. *Front Med (Lausanne)* 2024;11:1454150. doi: 10.3389/fmed.2024.1454150
13. Merle B, Haesebaert J, Bedouet A, et al. Osteoporosis prevention: Where are the barriers to improvement in French general practitioners? A qualitative study. *PLoS One* 2019;14(7):e0219681. doi: 10.1371/journal.pone.0219681
14. Tahmasbi F, Zabihiyeganeh M, Sadoughi F, et al. An insight for Fracture Liaison Service implementation: a qualitative study from a developing country. *Clin Rheumatol* 2025;44(1):503-14. doi: 10.1007/s10067-024-07208-8
15. Armstrong E, Yin X, Razee H, et al. Exploring barriers to, and enablers of, evidence-informed hip fracture care in five low- middle-income countries: China, India, Thailand, the Philippines and Vietnam. *Health Policy Plan* 2022;37(8):1000-11. doi: 10.1093/heapol/czac043
16. Edwards BJ, Bunta AD, Anderson J, et al. Development of an electronic medical record based intervention to improve medical care of osteoporosis. *Osteoporos Int* 2012;23(10):2489-98. doi: 10.1007/s00198-011-1866-9
17. Jensen CM, Smith AC, Overgaard S, et al. "If only had I known": a qualitative study investigating a treatment of patients with a hip fracture with short time stay in hospital. *Int J Qual Stud Health Well-being* 2017;12(1):1307061. doi: 10.1080/17482631.2017.1307061
18. Jensen CM, Overgaard S, Wiil UK, et al. Can Tele-Health Support Self-Care and Empowerment? A Qualitative Study of Hip Fracture Patients' Experiences With Testing an "App". *SAGE Open Nurs* 2019;5:2377960819825752. doi: 10.1177/2377960819825752

19. Narayanasamy M, Bishop S, Sahota O, et al. Acceptability and engagement amongst patients on oral and intravenous bisphosphonates for the treatment of osteoporosis in older adults. *Age Ageing* 2022;51(11) doi: 10.1093/ageing/afac255
20. Unson CG, Siccion E, Gaztambide J, et al. Nonadherence and osteoporosis treatment preferences of older women: a qualitative study. *J Womens Health (Larchmt)* 2003;12(10):1037-45. doi: 10.1089/154099903322643965
